# Effect of the intratumoral microbiota on spatial and cellular heterogeneity in cancer

**DOI:** 10.1038/s41586-022-05435-0

**Published:** 2022-11-16

**Authors:** Jorge Luis Galeano Niño, Hanrui Wu, Kaitlyn D. LaCourse, Andrew G. Kempchinsky, Alexander Baryiames, Brittany Barber, Neal Futran, Jeffrey Houlton, Cassie Sather, Ewa Sicinska, Alison Taylor, Samuel S. Minot, Christopher D. Johnston, Susan Bullman

**Affiliations:** 1 Human Biology Division, Fred Hutchinson Cancer Center, Seattle, WA USA; 2 University of Washington Medical Center, Seattle, WA USA; 3 Genomics Core, Fred Hutchinson Cancer Center, Seattle, WA USA; 4 Department of Pathology, Dana-Farber Cancer Institute, Boston, MA USA; 5 Herbert Irving Comprehensive Cancer Center, Columbia University, New York, NY USA; 6 Data Core, Fred Hutchinson Cancer Center, Seattle, WA USA; 7 Vaccine and Infectious Disease Division, Fred Hutchinson Cancer Center, Seattle, WA USA; 8 Present Address: Head and Neck Specialists, Sarah Cannon Cancer Institute, Charleston, SC USA

**Keywords:** Cancer microenvironment, Tumour heterogeneity, Oral cancer, Colorectal cancer, Pathogens

## Abstract

The tumour-associated microbiota is an intrinsic component of the tumour microenvironment across human cancer types^1,2^. Intratumoral host–microbiota studies have so far largely relied on bulk tissue analysis^1–3^, which obscures the spatial distribution and localized effect of the microbiota within tumours. Here, by applying in situ spatial-profiling technologies^4^ and single-cell RNA sequencing^5^ to oral squamous cell carcinoma and colorectal cancer, we reveal spatial, cellular and molecular host–microbe interactions. We adapted 10x Visium spatial transcriptomics to determine the identity and in situ location of intratumoral microbial communities within patient tissues. Using GeoMx digital spatial profiling^6^, we show that bacterial communities populate microniches that are less vascularized, highly immuno‑suppressive and associated with malignant cells with lower levels of Ki-67 as compared to bacteria-negative tumour regions. We developed a single-cell RNA-sequencing method that we name INVADEseq (invasion–adhesion-directed expression sequencing) and, by applying this to patient tumours, identify cell-associated bacteria and the host cells with which they interact, as well as uncovering alterations in transcriptional pathways that are involved in inflammation, metastasis, cell dormancy and DNA repair. Through functional studies, we show that cancer cells that are infected with bacteria invade their surrounding environment as single cells and recruit myeloid cells to bacterial regions. Collectively, our data reveal that the distribution of the microbiota within a tumour is not random; instead, it is highly organized in microniches with immune and epithelial cell functions that promote cancer progression.

## Abstract (web summary)

Spatial profiling and single-cell RNA sequencing are used to map the spatial distribution of the microbiota within human tumours, revealing how intratumoral microbial communities contribute to tumour heterogeneity and cancer progression.

## Main

In the tumours of patients with cancer, malignant cells are surrounded by a complex network of non-malignant cells that may have pro- or anti-tumorigenic effects depending on their cell type and abundance. In vitro and preclinical animal models indicate that bacteria in the tumour-associated microbiota have a role in cancer development^7^, metastasis^8–10^, immunosurveillance^11–13^ and chemoresistance^14,15^. There is strong molecular evidence of an intratumoral microbiota across at least 33 major cancer types^2,12,13,16^, as well as imaging data that show the co-localization of pan-bacterial markers with immune and epithelial cell targets, suggesting that the intratumoral microbiota can be intracellular^2,8,13^. However, the precise identity of these cell-associated organisms and the specific host cell types with which they interact in patient tumours have yet to be fully revealed. In addition, whether the spatial distribution of the intratumoral microbiota and specific host–microbial cellular interactions affect distinct functional capabilities within the tumour microenvironment (TME) is largely unknown. Here, focusing on cancers at the extremes of the gastrointestinal tract—oral squamous cell carcinoma (OSCC) and colorectal cancer (CRC)—we modify in situ spatial-profiling technologies and single-cell RNA sequencing (scRNA-seq) to concurrently map host–bacterial spatial, cellular and molecular interactions within the TME. Our results reveal how the intratumoral microbiota contributes to tumour heterogeneity.

## Heterogeneity of the intratumoral microbiota

**Extended Data Fig. 1:**
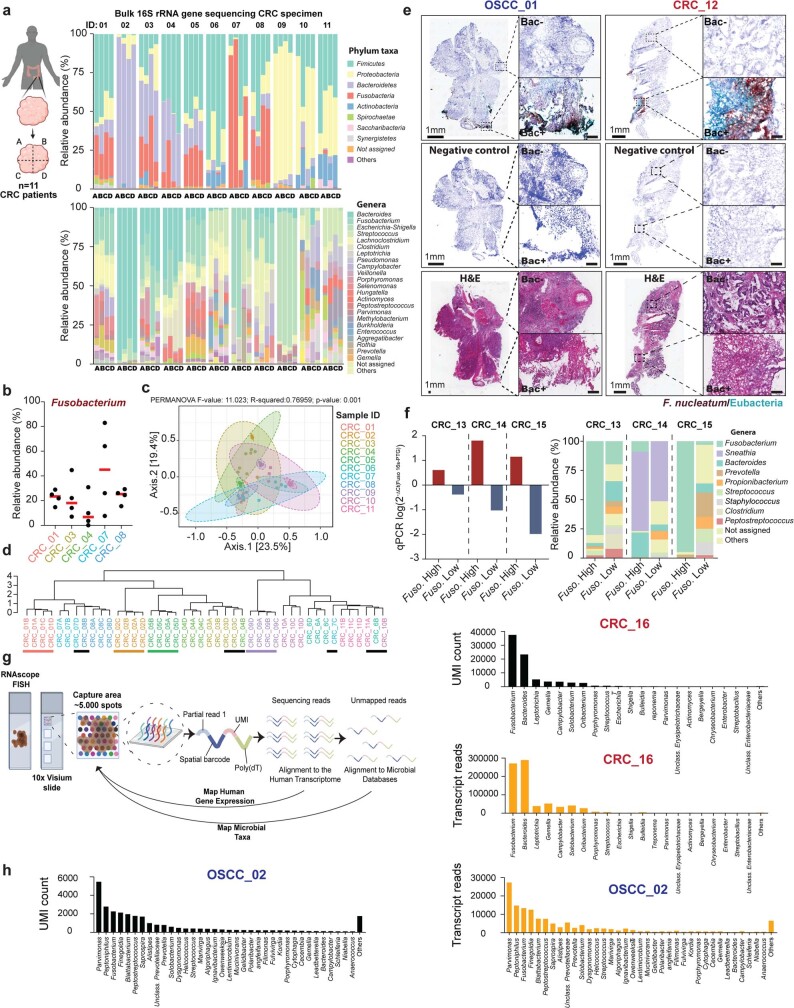
Heterogeneous distribution of the intratumoral microbiota throughout the tumour tissue. **a**, Relative abundance of bacterial communities at the phylum and genus level for each tumour piece (n = 4 per patient) from 11 human CRC tumour specimens, identified via bulk 16S rRNA gene amplicon sequencing. Tumour tissue pieces (n = 4) are denoted as A, B, C and D from the 11 patients with CRC as indicated. **b**, Relative abundance of *Fusobacterium* genera in each tumour piece from 5 positive CRC specimens described in (a). **c**, Principal component analysis (PCoA) plot representing beta diversity clustering (Bray-Curtis Index) of bacterial communities at the genus level from each piece of CRC tumour tissue and PERMANOVA analysis. **d**, Dendrogram representing clustering of the microbiome composition at genus level in the tumour pieces as described in (a). The index of dissimilarity between samples was calculated using the Bray-Curtis test. Hierarchical cluster analysis was performed to detect clustering of patient specimens (colour bars) by using the Ward clustering algorithm. **e**, Top: RNAscope-FISH images indicating the spatial distribution of *F. nucleatum* and other bacterial communities (eubacteria) across the tumour tissue from a OSCC and CRC specimen. *F. nucleatum* probe is red and eubacterial probe is cyan. Middle: RNAscope-FISH images showing the negative control staining for the images. Bottom: Haematoxylin and eosin staining (H&E) of the RNAscope images. **f**. Validation of RNAscope probes. Left: PCR quantifying *Fusobacterium nucleatum* 16S rRNA gene in macro-dissected tissue based on RNAscope probe binding containing relatively high (*Fuso*. High) or low (*Fuso*. Low) *F. nucleatum* positivity as it is indicated. Right: Microbiome analysis using 16S rRNA gene sequencing in tumour areas that are “*Fuso*. High” or “*Fuso*. Low” as it is indicated. **g**, Schematic showing the experimental approach: RNAscope imaging was implemented to identify tumour areas positive for bacteria or *F. nucleatum* from OSCC and CRC tumours embedded in OCT blocks. Tumour tissues were trimmed to fit the capture area (6.5 mm x 6.5 mm) on the 10x Visium slide. Following tissue permeabilization, RNA is released from cells and bind to an array of probes that are attached to the surface of the slide within capture spots. Each probe has a unique molecular identifier (UMI) and a barcode sequence providing the spatial coordinates for each transcript. cDNA is generated from the captured RNA through a reverse transcription reaction. The barcoded cDNA is denatured and pooled and then further processed to generate cDNA libraries. All transcripts are aligned against the human transcriptome to map the human gene-expression profile across the sample. The unmapped reads are then aligned against microbial databases through GATK PathSeq to identify the microbiome composition. **h**, Distribution of the bacterial UMI count and bacterial reads for top bacterial genera detected in 10x Visium data from the OSCC and CRC cases as it is indicated.

We performed 16S rRNA gene sequencing on 44 pieces of tissue from the tumours of 11 patients with CRC (Extended Data Fig. 1a), and observed that the composition of the intratumoral microbiota at the phylum and the genus level (Extended Data Fig. 1a and Supplementary Table 2), including *Fusobacterium* (Extended Data Fig. 1b), varied within individual patient tumours. Principal component analysis with beta diversity clustering (Extended Data Fig. 1c) and dendrogram analysis (Extended Data Fig. 1d) showed that over one third of the patients assessed (*n* = 4 out of 11) had relatively stable microbiome compositions; however, most patients (*n* = 7 out of 11) exhibited varying levels of heterogeneity in the intratumoral microbiome. This suggests a heterogeneous distribution of microorganisms in the tumour tissue in a subset of patients. Through targeted RNAscope–fluorescence in situ hybridization (RNAscope-FISH) imaging we visually confirmed the heterogeneous spatial distribution of these bacterial communities, including *Fusobacterium nucleatum*, for which both densely populated compartments of bacterial cell biomass and bacteria-negative regions are observed within the same tumour specimen (Extended Data Fig. 1e). The RNAscope approach was validated for *F.* *nucleatum* through quantitative PCR and microbiome analysis (Extended Data Fig. 1f).

**Fig. 1:**
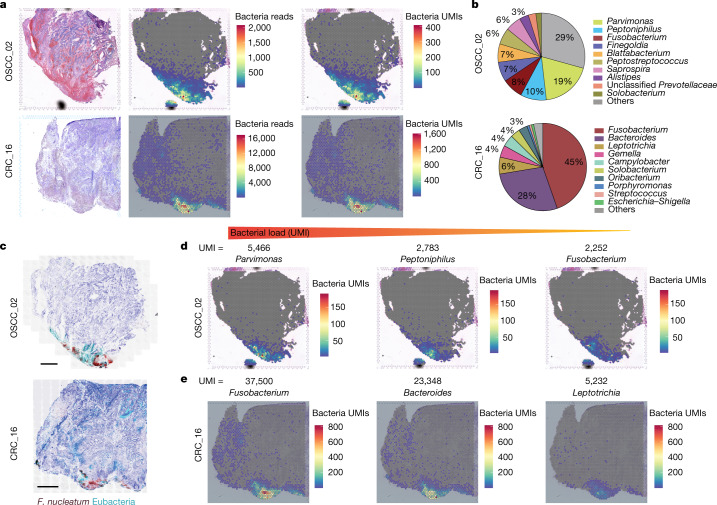
Assessing the spatial distribution of intratumoral bacteria throughout the tumour tissue. **a**, Haematoxylin and eosin (H&E) staining (left), spatial distribution of total bacterial reads (centre) and total UMI transcripts (right) throughout the tumour tissue in the 10x Visium capture slides from human OSCC and CRC specimens. **b**, Pie chart of the top 10 most dominant bacterial genera detected in the 10x Visium RNA-sequencing data from the OSCC and CRC tumours. **c**, RNAscope-FISH imaging showing the distribution of bacteria across the tumour tissue in a sequential slide following the 10x Visium section. The *F.* *nucleatum* probe is red and the eubacterial probe is cyan. Scale bars, 1 mm. **d**, Spatial distribution of *Parvimonas*, *Peptoniphilus* and *Fusobacterium* UMIs detected in the 10x Visium OSCC specimen data. **e**, Spatial distribution of *Fusobacterium*, *Bacteroides* and *Leptotrichia* UMIs detected in the 10x Visium CRC specimen data.

To gain further resolution on the spatial distribution and identity of the intratumoral microbiota, we applied an unbiased approach through 10x Visium spatial transcriptomics to a specimen of CRC and a specimen of OSCC (Extended Data Fig. 1g). After tissue processing, each captured microbial transcript, largely consisting of ribosomal RNA, was flanked with a barcode oligo sequence from the 10x Visium capture spot, providing spatial coordinates for the bacterial transcripts across the tumour tissue (Fig. 1a). In addition, the sequencing reads from individual microbial transcripts contained a unique molecular identifier (UMI), which enabled us to quantify the bacterial transcriptional load of viable organisms in these tissue sections (Fig. 1a). The resulting sequencing data were assessed using GATK PathSeq^17^ to taxonomically resolve in situ sequencing reads to the genus level (Fig. 1b and Supplementary Table 3). Within each block, a sequential tissue slide for targeted RNAscope-FISH confirmed the spatial distribution of bacteria within these tumours (Fig. 1c). Overall, bacterial transcripts were identified in 28% and 46% of the capture spots within OSCC and CRC tumours, respectively. When bacterial transcripts were detected, the number of different bacterial genera identified per capture spot ranged from 1 to 42 with a median of 8 in the OSCC tumour, and from 1 to 31 with a median of 2 in the CRC tumour. The UMI metric allowed the tissue transcriptional load of specific organisms to be quantified, and identified *Parvimonas*, *Peptoniphilus* and *Fusobacterium* as the most dominant genera in the OSCC tumour (Fig. 1d), and *Fusobacterium* and *Bacteroides* as the most dominant genera in the CRC tumour (Fig. 1e). Although a greater number of dominant genera were detected in the OSCC specimen (more than 1% relative abundance), the dominant genera in the CRC specimen (*Fusobacterium* and *Bacteroides*) had an order of magnitude more reads and UMIs than those in the OSCC specimen (Extended Data Fig. 1h and Supplementary Table 3). By applying and adapting this spatial transcriptomics approach to the intratumoral microbiota, we are able to directly identify, quantify and spatially map viable bacteria within histologically intact tumour tissues from patients. The detection of co-localized communities of both isolated genera and several different genera within capture spots highlights the complexity of intratumoral microbiota interactions across these tumour tissues.

## Intratumoral niches colonized by microorganisms

**Fig. 2:**
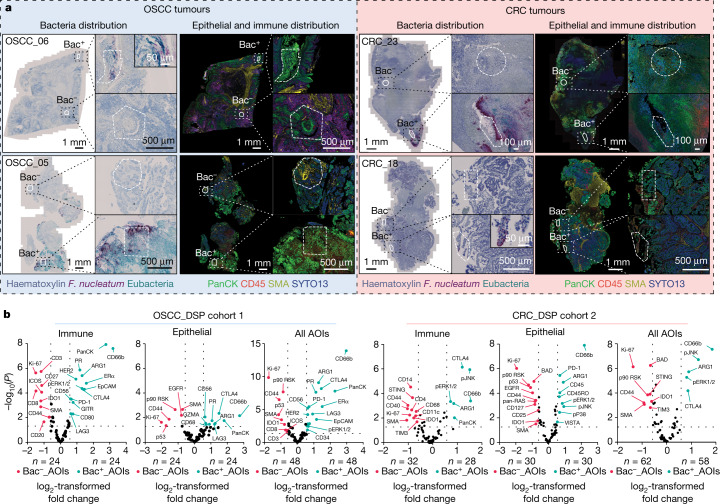
Evaluating the effect of the tumour-associated microbiota in local microniches. **a**, RNAscope-CISH images show the distribution of *F.* *nucleatum* (dark red) and other bacterial communities (eubacteria probe: cyan) in the tumour tissue; a sequential immunohistochemistry image shows the distribution of CD45^+^ (red) and PanCK^+^ (green) cells to identify the immune and epithelial compartments, respectively, in the tumour tissue. Inset images indicate representative AOIs that are positive and negative for bacteria and the corresponding UV exposure regions. **b**, Volcano plots from DSP data comparing the protein expression profiles in bacteria-positive AOIs and bacteria-negative AOIs from 8 OSCC (left) and 10 CRC (right) tumour specimens, referred to as microniche-level analysis. AOI comparative analysis, based on bacterial status, from immune (CD45^+^), epithelial (PanCK^+^) or combined (all AOIs) segmented data is shown. The number of AOIs per group is indicated. Dashed lines indicate the threshold of significant gene expression, defined as log_2_-transformed fold change ≥ 0.58 and ≤ −0.58 with −log_10_(*P*) ≥ 1.301 after linear mixed effect model (LMM) analysis and Benjamini–Hochberg multiple-correction testing. The p prefix indicates phosphorylation; ERK1/2 refers to ERK1 and ERK2; PR, progesterone receptor.

**Extended Data Fig. 2:**
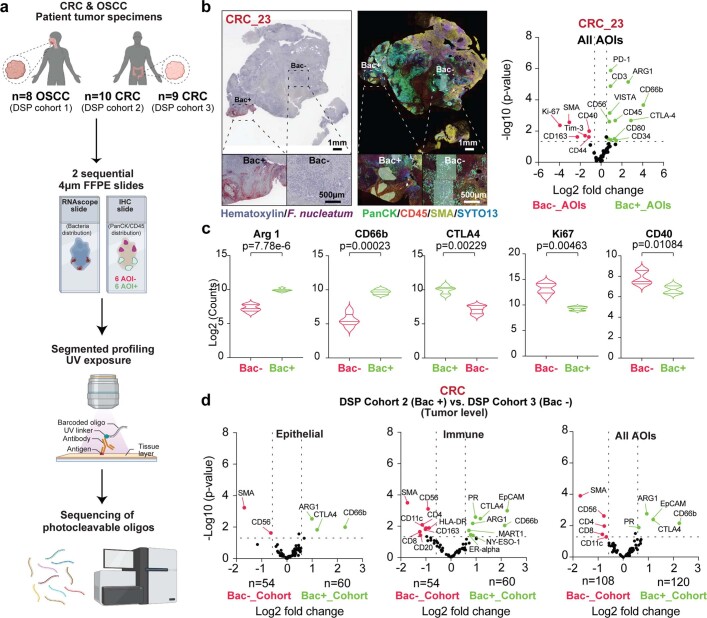
The tumour-associated microbiota resides in highly immunosuppressive microniches with a low proliferation rate. **a**, Experimental approach: GeoMx DSP was implemented to assess bacteria-associated microniches in one OSCC DSP cohort (n = 8) and two CRC DSP cohorts (RNAscope bacteria-positive cohort n = 10 and RNAscope bacteria-negative cohort n = 9). Sequential 4 µm-FFPE slides were prepared to identify spatial bacterial tumour distribution (RNAscope-CISH using *F. nucleatum* and eubacteria probes) and immunohistochemistry for immune (CD45+) and epithelial (PanCK+) compartments on the DSP slide treated with the 77-antibody panel. Segmented profiling for CD45+ and PanCK+ was performed on bacteria-positive AOIs (AOI_bac+) and bacteria-negative AOIs (AOI_bac-) per specimen, releasing photocleavable barcoded oligos for sequencing. Sequenced oligos provided the spatial information of the respective protein target in the bacteria positive or negative regions. **b**, RNAscope-CISH (left) showing the distribution of *F. nucleatum* (dark red), throughout the tumour tissue from a CRC specimen. A sequential slide (right) showed the distribution of the immune (CD45+; red) and epithelial (PanCK+; green) compartments by IHC staining. Inset images indicated the AOIs that were selected for DSP analysis from a bacteria-positive (Bac+) and bacteria-negative (Bac-) regions as it is indicate. Volcano plot showed the differential expression of genes from a single CRC sample comparing Bac+ with Bac- regions from the same tissue sample. Dashed lines indicate the threshold of significant gene expression defined as the Log2 fold change ≥0.58 and ≤−0.58 with a -Log10 p value ≥1.301 following LMM analysis and Benjamini–Hochberg multiple-correction testing. A 52-antibody panel were included here (this did not include the Cell Death and MAPK modules applied to DSP cohorts 1—3). **c**, Violin plots demonstrate the immuno‑suppressive microenvironment in bacteria-positive regions (Bac+) from the sample described in (b), highlighting the upregulation of ARG1 and CTLA4 and the enrichment of myeloid CD66b+ cells with lower expression of Ki67 and the T cell co-stimulatory molecule CD40 compared to bacteria-negative regions. p values calculated by t-test. **d**, Volcano plots indicate the differential gene-expression profile using the GeoMx DSP platform comparing AOIs from tumours (DSP cohort 2) that were RNAscope bacteria positive (Bac+; n = 120) against AOIs from tumours (DSP cohort 3) that were RNAscope bacteria negative (Bac-; n = 108). Using segmented analysis, the barcode oligos were collected either from the immune (CD45+) segment, epithelial (PanCK+) segment or both (All AOIs). Dashed lines indicate the threshold of significant gene expression defined as the Log2 fold change ≥0.58 and ≤−0.58 with a -Log10 p value ≥1.301 following LMM analysis and Benjamini–Hochberg multiple-correction testing.

Given that the intratumoral microbiota has a heterogeneous distribution within individual tumour tissues, we sought to determine whether this spatial distribution correlated with distinct functions within the TME. Using a targeted approach through the GeoMx digital spatial profiling (DSP) platform (Extended Data Fig. 2a), we quantified the expression profile of 77 proteins that are associated with anti-tumour immunity and cancer progression. Segmented profiling was implemented to enrich the extracted protein data from either immune or epithelial cancer compartments within tissue areas of interest (AOIs), which were annotated by RNAscope–chromogenic in situ hybridization (RNAscope-CISH) as positive or negative for bacteria (Bac^+^ or Bac^−^, respectively; Fig. 2a,b).

Within CD45^+^ immune compartments of both OSCC (DSP cohort 1; *n* = 8 patients) and CRC (DSP cohort 2; *n* = 10 patients) tumours, we independently show that bacteria reside in highly immunosuppressive microniches that are characterized by an enrichment of mature CD66b^+^ myeloid cells along with an upregulation of the immunosuppressive molecule ARG1 (arginase 1) and the immune checkpoint protein CTLA4 (cytotoxic T-lymphocyte-associated protein 4) (Fig. 2b). In addition, in both cancer types, we detected increased levels of phosphorylated ERK1 and ERK2 (Fig. 2b), which suggests that the myeloid response against intratumoral bacteria might occur through activation of the MAPK signalling pathway^18^. In OSCC tumours, the T-cell-inhibitory receptor PD-1 was overexpressed in bacteria-positive microniches as compared to bacteria-negative areas within specimens (Fig. 2b). This corresponded to a relative downregulation of T cell markers such as CD3, CD8, CD4, CD27 and CD44 in both the OSCC and the CRC cancer tissue, along with reduced expression of the proliferation marker Ki-67, and suggests that T cells are excluded in bacteria-colonized regions of these two cancers of the gastrointestinal tract (Fig. 2b and Supplementary Table 4).

**Extended Data Fig. 3:**
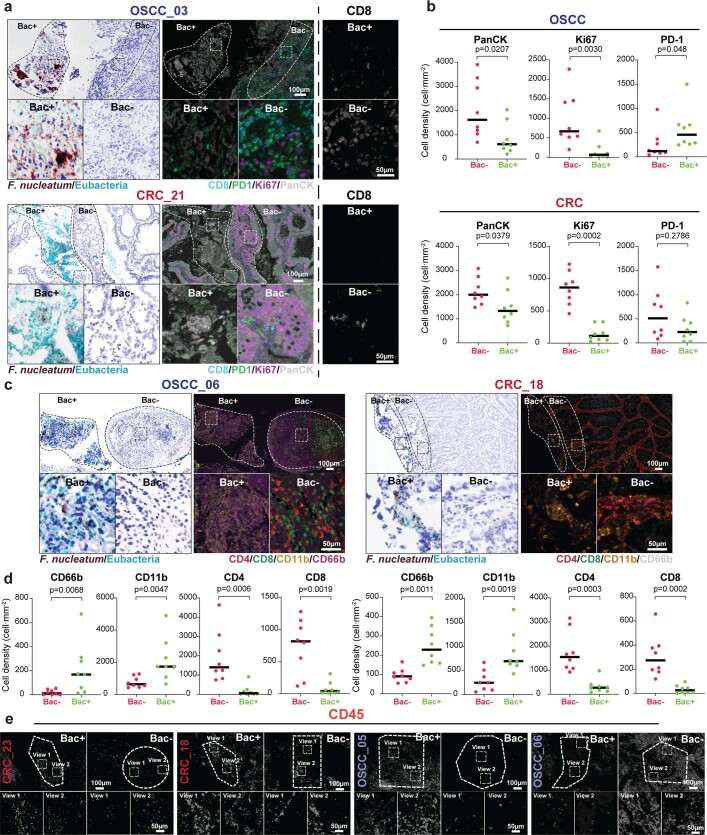
The tumour-associated microbiota resides in T-cell-excluded areas, with lower proliferation capabilities. **a**, RNAscope-FISH images showing bacteria-positive regions (Bac+) with the corresponding adjacent bacteria-negative region (Bac-) from a OSCC and CRC sample. A sequential IHC slide indicates the staining of CD8, PD-1, PanCK and Ki-67 in Bac+ and Bac- regions from the same tumour samples as it is indicated. Right panels indicate the CD8 fluorescent signal from a OSCC and CRC case comparing Bac+ vs Bac- regions from the same tumour tissue. **b**, Quantification of cell densities for PanCK, Ki-67 and PD-1, expressing cells in bacteria-positive regions in comparison to the contiguous bacteria-negative regions. p-values were calculated by Mann–Whitney test **c**, Representative RNAscope-FISH images showing bacteria-positive regions (Bac+) with the corresponding adjacent bacteria-negative region (Bac-) from a OSCC and CRC sample (dashed areas within images). A sequential IHC slide indicates the distribution of immune cell populations including myeloid CD66b+ or CD11b+ cells and CD4+ or CD8+ T cells. Magnified/inset images show the immune cell population that is more abundant in bacteria-positive (Bac+) and bacteria-negative (Bac-) regions for each tumour sample as it is indicated. **d**, Quantification of cell densities of CD66b, CD11b, CD4 and CD8 expressing cells in Bac+ regions compared to the contiguous Bac- region from the same (n = 4) OSCC (left) and (n = 4) CRC (right) tumour samples in two separate field of views. p-values were calculated by Mann–Whitney test. **e**, IHC images showing the CD45 fluorescent signal from the tumour samples described in Fig. 2a.

In the PanCK^+^ epithelial tumour compartment of both cancer types, bacteria-colonized regions were less vascularized than bacteria-negative regions, with reduced expression of smooth muscle actin (SMA) and lower levels of proliferation, as characterized by the downregulation of Ki-67 and p90 RSK (ref. ^19^) (Fig. 2b). In bacteria-colonized microniches of both OSCC and CRC tumour tissue, we detected a significant reduction in the protein expression of the wild-type configuration of the tumour suppressor p53, indicating that bacterial localization correlates with highly transformed cancer cells within the TME (Fig. 2b). Furthermore, bacteria-colonized microniches had significantly increased levels of phosphorylation of JNK, ERK1 and ERK2 and P38 in CRC tumours, thus revealing signalling pathways that are activated in response to bacteria (Fig. 2b). When applied to a single tumour specimen from a patient with CRC (CRC_23) using all 24 AOIs, similar protein expression profiles were obtained (Extended Data Fig. 2b,c and Supplementary Table 4). The combination of RNAscope and immunohistochemistry (IHC) techniques supported the findings from DSP of an overexpression of PD-1 in bacteria-positive microniches in OSCC specimens, in addition to a significant reduction in the levels of Ki-67, suggesting that infected regions of the OSCC and CRC tumour tissue have a lower proliferation potential than uninfected regions (Extended Data Fig. 3a,b).

Further analysis was performed in which we compared CRC tumours that have detectable bacteria (DSP cohort 2; *n* = 10 patients) with CRC tumours that were negative for bacteria by RNAscope analysis (DSP cohort 3; *n* = 9 patients), to determine whether bacteria-colonized microniches could have a broader effect at the tumour tissue level. We found that bacteria-positive tumours showed reduced expression levels of CD4 and CD8, along with an increased expression of immunosuppressive molecules such as CTLA4 and ARG1, and an enrichment of CD66b^+^ myeloid cells (Extended Data Fig. 2d and Supplementary Table 4), supporting previous bulk tissue analysis^20,21^.

RNAscope and IHC confirmed that bacteria-positive regions of tissue had significant increases in CD11b^+^ and CD66b^+^ myeloid cells, along with lower densities of CD4^+^ and CD8^+^ T cells, as compared to immediately adjacent bacteria-negative regions; this indicates that the effect of the tumour-associated microbiota is highly localized (Extended Data Fig. 3c,d).

## Microorganism-driven single-cell heterogeneity

**Extended Data Fig. 4:**
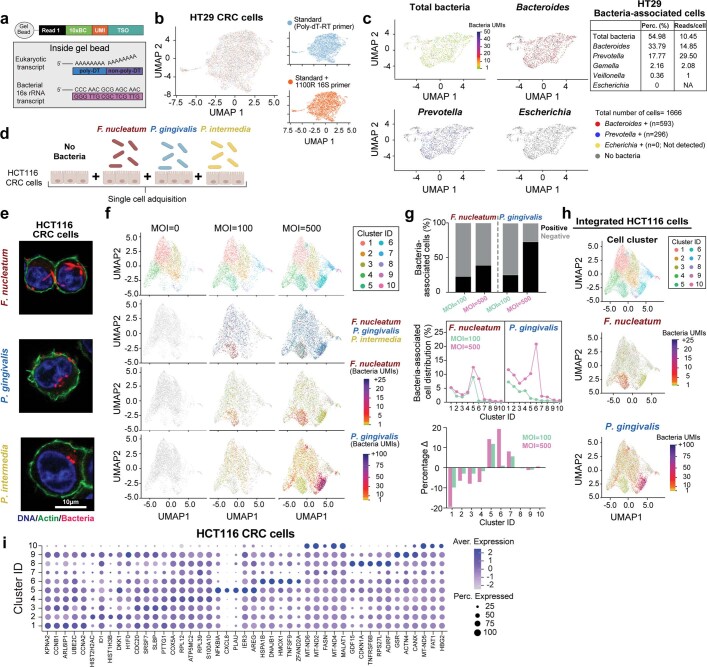
Detection of bacteria-associated single cells using the INVADEseq technique. **a**, Schematic showing a modified gel bead emulsion (GEMs) by introducing a primer (1100R 16S) that targets a conserved region of the bacterial 16S rRNA, thus allowing cDNA generation of bacterial transcripts from the associated human single cells. In addition to the standard 10x genomics 5’ library preparation, bacterial cDNA was amplified with a nested conserved 16S rRNA gene primer and size selected libraries were sequenced and assessed through GATK PathSeq to identify bacterial taxa. Sequencing reads from the 16S rRNA amplified libraries retain the 10x genomics barcode sequence which facilitated mapping of annotated bacterial reads directly to the host single cells they are associated with. **b**, UMAP plots showing single-cell transcriptome of HT-29 cells with (orange dots) and without (blue dots) the 1100R 16S primer in the amplification mix before single-cell cDNA generation. UMAP plot inserts show the transcriptome for each condition, indicating no differences in the human gene-expression profile when the 1100R 16S primer was added. **c**, UMAP plots indicating single-cell transcriptome of HT-29 cells co-incubated with *Bacteroides fragilis*, *Prevotella intermedia*, *Gemella haemolysans*, *Veillonella parvula* and *Escherichia coli DH5a* for 3 h at MOI = 100. Insert table indicates the percentage of bacteria-associated single cells and total bacterial reads per cell per bacterial taxa. Note: *Escherichia coli DH5a* reads were not detected in human single cells. **d**, Experimental approach: HCT116 cancer cells were co-culture with either *Fusobacterium nucleatum*, *Porphyromonas gingivalis* or *Prevotella intermedia* at total multiplicity of infection (MOI) of 0, 100 and 500 for 3 hrs and processed for INVADEseq. **e**, Confocal images showing intracellular bacteria in HCT116 cancer cells after 3 h of incubation with *F. nucleatum*, *P. gingivalis* and *P. intermedia* as it is indicated. **f**, From top to bottom, UMAP plots from scRNA-seq data showing: Cell cluster distribution based on epithelial cell transcription, infected HCT116 cancer cells with *F. nucleatum*, *P. gingivalis* and *P. intermedia*, and expression level (UMI, bacterial load) of *F. nucleatum* and *P. gingivalis* transcripts in cancer cells following bacteria treatment for 3 h at multiplicity of infection (MOI) 0, 100 and 500 as it is indicated. Cluster ID indicates a unique transcriptional cellular group predicted by Seurat package (See methods). Colour bars indicate the expression level (UMI counts) of *F. nucleatum* and *P. gingivalis* transcripts as it is indicated. **g**, Top: Percentage of bacteria-associated cells positive for either *F. nucleatum* or *P. gingivalis* at MOI = 100 and 500 as it is indicated. Middle: Distribution of *F. nucleatum* and *P. gingivalis-*associated cells across all cell cluster annotated in (f) whereby all cell clusters combined, bacteria positive and negative, equal 100%. Bottom: Relative change in the percentages (Δ%) of cancer epithelial cell clusters between bacteria-associated cancer cells at MOI = 100 or 500 compared to the untreated control cell population (MOI = 0) for each cancer cell cluster annotated in (f). **h**, UMAP plots showing cancer epithelial cell clusters and detection of *F. nucleatum* and *P. gingivalis* transcripts following data integration from the experimental conditions described in (d). **i**, Dot plot showing the relative expression of gene markers for each ID cluster from CRC epithelial cells derived from the HCT116 cell line. Colour bars indicate the average expression level, and the dots represent the percentage expression level for each gene marker.

The presence of bacteria within individual host cells of the TME has been reported across a range of human cancer types^2,13^. However, we have little information on the identity of invasive bacteria, the host cells that they interact with and how these host–bacterial associations affect cellular function within the TME. To investigate bacterial–host cell-to-cell interaction within the TME and the effect on host cell transcriptomics, we developed INVADEseq (invasion–adhesion-directed expression sequencing) by introducing a primer that targets a conserved region of bacterial 16S rRNA, facilitating the generation of cDNA libraries with bacterial transcripts from the bacteria-associated human cells (Extended Data Fig. 4a). Addition of this bacteria-targeting primer did not affect the gene-expression profile of human CRC cells (Extended Data Fig. 4b), and validation co-culture experiments with non-adherent and non-invasive *Escherichia coli* DH5α (Extended Data Fig. 4c) showed specificity for cell-associated bacteria.

To further validate this approach, the human CRC cell line HCT116 was infected with three invasive bacterial species—*F.* *nucleatum*, *Porphyromonas gingivalis* and *Prevotella intermedia*—at a multiplicity of infection (MOI) of 100:1 and 500:1, and processed for INVADEseq (Extended Data Fig. 4d). Confocal imaging indicated the presence of intracellular bacteria in cancer cells after bacterial co-culture (Extended Data Fig. 4e). Using INVADEseq, we mapped bacterial reads to single human cells (Extended Data Fig. 4f,g). At the cell-cluster level for these epithelial single cells (clusters 1–10), most *F.* *nucleatum*- and *P.*  *gingivalis*-positive single cells were distributed in cancer cell clusters 5 and 6, respectively (Extended Data Fig. 4g). Both cell clusters (clusters 5 and 6) were very minor cell populations in the uninfected control group (Extended Data Fig. 4f). When compared to uninfected controls (MOI = 0), the appearance of cell clusters 5 (*Fusobacterium* cluster) and 6 (*Porphyromonas* cluster) coincided with a relative reduction in the percentage of cluster 1 (uninfected control cluster) (Extended Data Fig. 4g). This finding suggests that *F.* *nucleatum* and *P.* *gingivalis* affect cancer cell heterogeneity by altering distinct transcriptional programs that contribute to specific cell clusters (Extended Data Fig. 4g).

**Extended Data Fig. 5:**
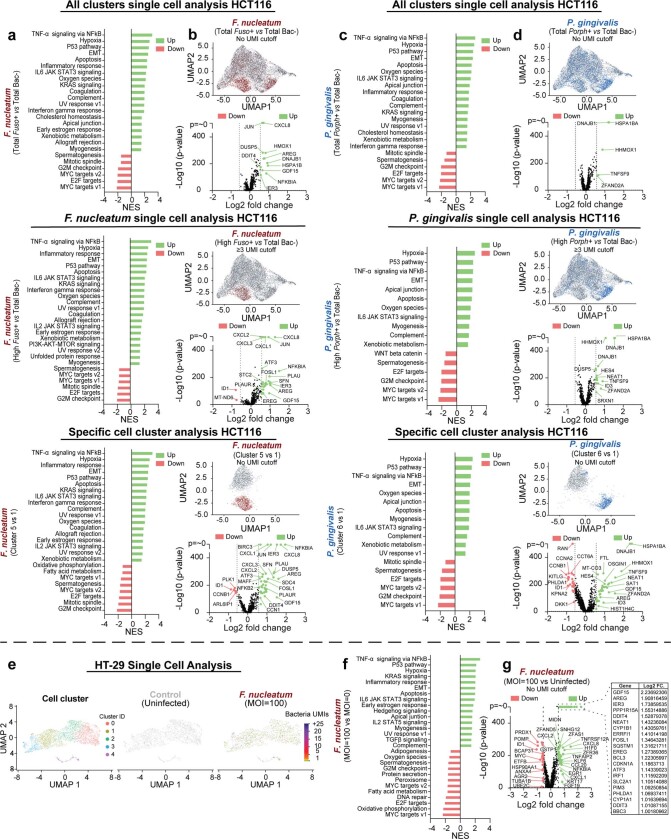
Differential gene expression and GSEA analysis comparing distinct CRC single-cell groups on the basis of bacteria association. **a**, GSEA analysis indicating the signalling pathways that are differentially regulated in HCT116 cells co-incubated with *Fusobacterium nucleatum* at MOI = 500 for 3 h between different single-cell groups as follow: Top: Total *F. nucleatum*-associated cells (Total *Fuso+*) compared to total bacteria-negative cells (Total Bac-) in the entire sample “All clusters single cell analysis”. Middle: *F. nucleatum*-associated cells that contain ≥3 *F. nucleatum* UMIs (High *Fuso*) compared to total bacteria-negative cells (Total Bac-) in the entire sample “*F. nucleatum* single cell analysis”. Bottom: *F. nucleatum*-associated cells from cell cluster 5 compared to bacteria-negative cells from cluster 1 “Specific cell cluster analysis”. **b**, UMAP plots showing the cell population that are highlighted for each analysis described in (a), in which the *F. nucleatum*-associated cells are coloured in red and the bacteria-negative cells are coloured in grey as it is indicated. Volcano plots indicate the differential gene-expression profile between the cell populations described in (a). Dashed lines indicate the threshold of significant gene expression defined as the Log_2_ fold change ≤−0.58 and ≥0.58 with a -Log_10_ p value ≥1.301. **c**, GSEA analysis indicates the signalling pathways that are differentially regulated in HCT116 cells co-incubated with *Porphyromonas gingivalis* at MOI = 500 for 3 h between different cellular groups as follow: Top: Total *P. gingivalis* -associated cells (Total *Porph+*) compared to total bacteria-negative cells (Total Bac-) in the entire sample “All clusters single cell analysis”. Middle: *P. gingivalis* -associated cells that contain ≥3 *P. gingivalis* UMIs (High *Porph*) compared to total bacteria-negative cells (Total Bac-) in the entire sample “*P. gingivalis* single cell analysis”. Bottom: *P. gingivalis*-associated cells from cell cluster 6 compared to bacteria-negative cells from cluster 1 “Specific cell cluster analysis”. **d**, UMAP plots show the cell populations that are highlighted for each analysis described in (c), in which the *P. gingivalis*-associated cells are coloured in blue, and the bacteria-negative cell population are coloured in grey as it is indicated. Volcano plots indicate the differential gene-expression profile between the cell populations described in (c). Dashed lines indicate the threshold of significant gene expression defined as the Log_2_ fold change ≤−0.58 and ≥0.58 with a -Log_10_ p value ≥1.301. **e**, UMAP plots showing cell cluster distribution and the detection of bacterial transcripts in HT-29 cancer cells treated with or without *F. nucleatum* at MOI of 100 for 2 h. Colour bar indicates expression level (UMI counts) of *F. nucleatum*. **f**, GSEA analysis indicating the signalling pathways that are differentially regulated in HT-29 cancer cells treated with *F. nucleatum* compared to an uninfected control cancer cell population. **g**, Volcano plot showing the gene-expression profile in HT-29 cells treated with *F. nucleatum* relative to bacteria-negative cells. Dashed lines indicate the threshold of significant gene expression defined as the Log2 fold change ≥0.58 and ≤−0.58 with a -Log10 p value ≥1.301. Wilcoxon Rank Sum test was implemented to calculate the normalized enrichment score (NES) in panels (a), (c) and (f). A LMM analysis followed by Benjamini–Hochberg multiple-correction test was used to calculate the fold change and p-values for each gene in panels (b), (d) and (g).

After integrating data from the three HCT116 samples (Extended Data Fig. 4h,i), we compared the gene expression of *F.* *nucleatum*- or *P.* *gingivalis*-associated single epithelial cells to that of the bacteria-negative epithelial single cells (Total Bac^−^). We noted that the number of differentially expressed genes increased when a bacterial UMI cut-off (≥3), a proxy for bacterial transcriptional load, was applied (Extended Data Fig. 5a–d and Supplementary Table 5). Furthermore, a comparison of cells from cluster 5 (*Fusobacterium* cluster) and cluster 6 (*Porphyromonas* cluster) to bacteria-negative cells from cluster 1 (uninfected control cluster) showed that bacteria-infected cells exhibited a significant upregulation of signalling pathways that are involved in the response to bacterial infection, such as the TNF pathway and pathways related to inflammation and hypoxia, as well as cancer cell progression via the epithelial–mesenchymal transition (EMT) and the p53 signalling pathway^22,23^. Bacteria-infected cells also showed a downregulation of cell-cycle signalling pathways that relate to the formation of the mitotic spindle and the G2–M DNA damage checkpoint, as compared with cells from the uninfected control cluster (Extended Data Fig. 5a–d). At the gene-expression level, bacteria-associated single epithelial cells showed significant increases in the expression of molecules that are positively associated with metastasis, such as *PLAU*, *PLAUR*, *RELB* and *AREG*, along with an upregulation of the chemokines *CXCL1*, *CXCL2*, *CXCL3* and the neutrophil chemoattractant *CXCL8*, along with members of the TNF family (Extended Data Fig. 5a–d). We also noted a significant upregulation of transcription factors including *NFKBIA*, *NFKB2*, *NEAT1*, *SAT1* and members of the JUN and FOS family, with a downregulation of the cyclins *CCNB1* and *CCNA*2 (Extended Data Fig. 5a–d). Similar findings were observed when CRC-derived HT-29 cells were treated with *F.* *nucleatum* at a MOI of 100:1; that is, an increase in the expression of genes that encode molecules related to inflammation through TNF, hypoxia, the EMT and p53 signalling pathways, and a reduction in the expression of genes that are involved in DNA repair (Extended Data Fig. 5e–g and Supplementary Table 5).

**Fig. 3:**
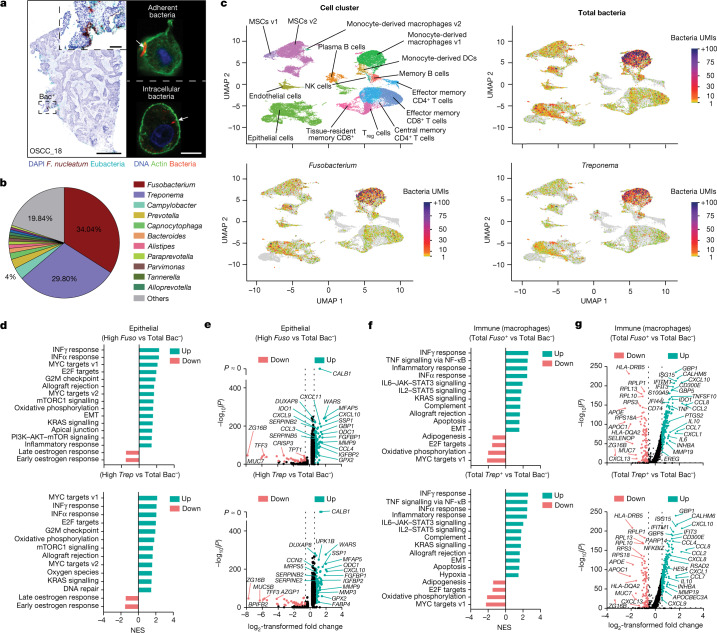
Effect of cell-associated intratumoral bacteria on transcriptomics in host single cells. **a**, RNAscope-FISH (left) shows the distribution of intratumoral bacteria in a tumour from a patient with OSCC. Confocal images (right) show bacteria-associated single cells after tissue dissociation. Scale bars, 1 mm (left); 5 μm (right). **b**, Microbiome composition at the genus level after integration of tumour scRNA-seq data from seven patients with OSCC using the INVADEseq method. **c**, UMAP plots indicate host cell annotation and bacteria transcripts (UMI) from total bacteria and *Fusobacterium*- and *Treponema*-associated cells in integrated tumour single-cell data from seven patients with OSCC as indicated. Colour bars indicate the bacterial UMI transcripts for total bacteria and for each bacterial species as indicated. DCs, dendritic cells; MSCs, mesenchymal stem cells; T_reg_ cells, regulatory T cells. **d**, GSEA analysis showing the signalling pathways that are differentially regulated in cells that contain ≥3 *Fusobacterium* UMI (High *Fuso*) or ≥3 *Treponema* UMI (High *Trep*) transcripts versus (vs) total bacteria-negative cells (Total Bac^−^) from the epithelial cell cluster. **e**, Volcano plots showing the differentially expressed genes between cell populations described in **d**. Dashed lines indicate the threshold of significant gene expression, defined as log_2_-transformed fold change ≤ −0.58 and ≥ 0.58 with −log_10_(*P*) ≥ 1.301. **f**, GSEA analysis showing the signalling pathways that are differentially regulated between total *Fusobacterium* (Total *Fuso*^+^) or total *Treponema* (Total *Trep*^+^) associated cells versus bacteria-negative cells (Total Bac^−^) in the monocyte-derived macrophage-v1 cell cluster. **g**, Volcano plots showing the differentially expressed genes between cell populations described in **f**. Dashed lines indicate the threshold of significant gene expression, defined as log_2_-transformed fold change ≤ −0.58 and ≥ 0.58 with −log_10_(*P*) ≥ 1.301. The normalized enrichment scores (NESs) in **d**,**f** were calculated using the Wilcoxon rank sum test. LMM analysis followed by Benjamini–Hochberg multiple-correction testing was used to calculate the fold change and *P* values for each gene in **e**,**g**.

**Extended Data Fig. 6:**
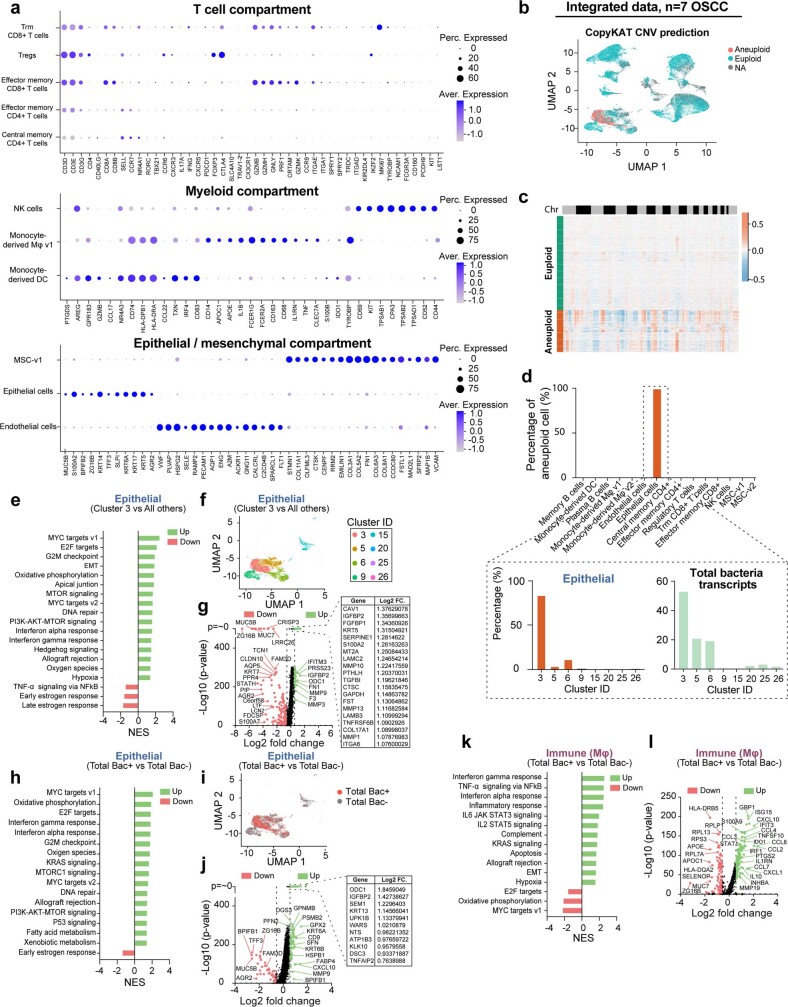
Bacteria-associated single cells correlate with upregulation of cell inflammation and cancer progression pathways in patients with OSCC. **a**, Dot plot showing the relative expression of gene markers for the T cell, myeloid and epithelial / mesenchymal cell compartment from single-cell data from patients with OSCC (n = 7 patients) as it is indicated in the cell clusters annotated in the UMAP plot from Fig. 3c. Colour bars indicate the average expression level, and the dots represent the percentage of expression for each gene marker. **b**, UMAP plot indicates the distribution of aneuploid and euploid cells in samples from patients with OSCC. **c**, Heat map shows the copy number variations (CNV) across 41,723 cells from the epithelial cell cluster as it is indicated. **d**, Percentage of aneuploid cells for each cell type annotated in Fig. 3c. Insert tables indicate the percentage of aneuploid cells and bacterial transcripts (UMIs) for each sub-cell clusters in the epithelial cell cluster as it is indicated. **e**, GSEA analysis showing the signalling pathways that are differentially regulated in cells from the sub-cell cluster 3, which contains the majority of aneuploid cancer cells, relative to other sub-cell clusters (All others) from the epithelial cell cluster. **f**, UMAP plot highlighting the epithelial sub-clusters that are contained in the epithelial cell cluster detected in Fig. 3c. **g**, Volcano plot showing the gene-expression profile in cancer cells from sub-cell cluster 3 in comparison to other sub-cell clusters from the epithelial cell cluster. Dashed lines indicate the threshold of significant gene expression defined as the Log2 fold change ≥0.58 and ≤−0.58 with a -Log10 p value ≥1.301. **h**, GSEA analysis showing the signalling pathways that are differentially regulated comparing total bacteria-associated cells (Total Bac+) vs bacteria-negative cells (Total Bac-) in the epithelial single cells as described in Fig. 3c. **I**, UMAP plot highlighting the transcriptional cellular group that is associated with bacterial invasion (Total Bac+; red cells) along with the bacteria-negative cell population (Total Bac-; grey cells). **j**, Volcano plot indicates the gene-expression profile in total bacteria-associated cell (Total Bac+) relative to total bacteria-negative cells (Total Bac-) in the epithelial single-cell cluster described in Fig. 3c. **k**, GSEA analysis showing the signalling pathways that are differentially regulated comparing total bacteria-associated cells (Total Bac+) vs bacteria-negative cells (Total Bac-) in the macrophage single cells as described in Fig. 3c. **l**, Volcano plot indicates the gene-expression profile in total bacteria-associated cell (Total Bac+) relative to total bacteria-negative cells (Total Bac-) in the macrophage cell cluster described in Fig. 3c. Dashed lines indicate the threshold of significant gene expression defined as the Log2 fold change ≥0.58 and ≤−0.58 with a -Log10 p value ≥1.301. Wilcoxon Rank Sum test was implemented to calculate the normalized enrichment score (NES) in panels (e), (h) and (k). A LMM analysis followed by Benjamini–Hochberg multiple-correction test was used to calculate the fold change and p-values for each gene in panels (g), (j) and (l).

The INVADEseq method was subsequently implemented to examine bacteria–host interactions in fresh tumour tissues from seven patients with OSCC. After the tissues were dissociated to single cells, confocal imaging showed that single cells from a tumour from a patient with OSCC contained cell-adherent and intracellular bacteria (Fig. 3a). Integrated scRNA-seq from the seven tumours revealed that the intratumoral microbiota is dominated by bacterial species that belong to the *Fusobacterium* (34%) and *Treponema* (29.8%) genera (Fig. 3b). Mapping bacterial reads from INVADEseq analysis to annotated single cells showed that *Fusobacterium* and *Treponema* were predominantly associated with the epithelial and monocyte-derived macrophage-v1 (referred to as the macrophage cluster) cell clusters in these patient tumours, with a total bacterial infection rate of 25% and 52%, respectively (Fig. 3c and Extended Data Fig. 6a). INVADEseq cannot distinguish whether bacteria are actively invading the macrophage cells or whether the macrophages are phagocytizing the bacteria; however, we refer to these cells as ‘macrophages with bacteria engulfed’. Within the epithelial cell clusters, cells in cluster 3 were identified as aneuploid, confirming that these are tumour cells with severe chromosomal instability (Extended Data Fig. 6b–d). Notably, this aneuploid epithelial cell cluster contained most of the bacterial UMI transcripts, as compared to other euploid epithelial cell clusters (Extended Data Fig. 6d). Gene set enrichment analysis (GSEA) confirmed that the cells from the bacteria-dominant epithelial cell cluster 3 were indeed cancer cells, with gene-expression signatures characterized by an upregulation of signalling pathways involved in cancer progression, including EMT, PI3K–AKT–mTOR, hypoxia and the interferon (IFN) response, among others (Extended Data Fig. 6e–g).

To determine whether the dominant cell-associated bacterial genera, *Fusobacterium* and *Treponema*, affected epithelial signalling pathways, *Fusobacterium*- or *Treponema*-associated single cells (UMI ≥ 3) were compared to bacteria-negative cells (Total Bac^−^) from the epithelial cell cluster. After GSEA analysis, we observed a significant upregulation of IFN and JAK–STAT signalling, with increased expression of molecules from the SERPIN family; chemokines such as *CXCL10*, *CXCL11*, *CCL4* and *CCL3*; and metalloproteinases, including *MMP9* and *MMP3* (Fig. 3d,e and Supplementary Table 6). A comparison of general bacteria-positive epithelial cells (Total Bac^+^), independent of a specific genus, and bacteria-negative cells (Total Bac^−^) showed that gene expression and cell signalling pathways related to cancer progression were  modestly affected in bacteria-positive epithelial cells, as compared to the effects that were observed in cells infected with specific taxa (Extended Data Fig. 6h–j and Supplementary Table 6). This is likely to be reflective of taxa-specific epithelial cell interactions or capabilities rather than a general bacteria-induced response.

**Extended Data Fig. 7:**
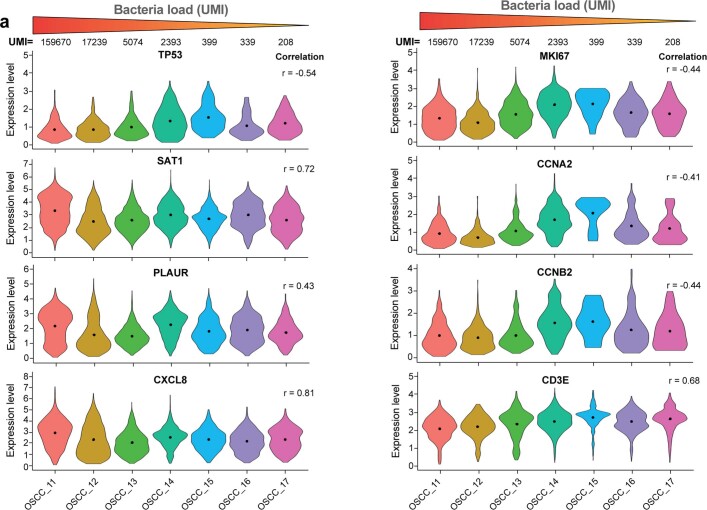
Expression levels of genes that are correlated or anticorrelated with bacterial load (UMI) in patients with OSCC. **a**, Expression level of genes as a function of single-cell-associated bacterial load (UMI) in seven patients with OSCC. The colour bars (top) indicate a gradient of bacterial load across the samples; the bacterial UMI values are designated for each patient. The correlation values were measured for each gene based on mean gene expression and bacterial UMI counts as it is indicated.

At the specimen level, the total bacterial load from each sample was negatively correlated with the expression of *TP53* and positively correlated with its negatively regulated target molecule, *SAT1* (Extended Data Fig. 7a)—consistent with our findings from DSP (Fig. 2), in which bacteria colonized microniches with reduced levels of wild-type p53. In addition, the total bacterial load negatively correlated with the expression of the proliferation marker *MKI67*, which encodes Ki-67 (Extended Data Fig. 7a), providing support for our spatial microniche data (Fig. 2 and Extended Data Fig. 3a,b).

In the macrophage cell cluster, by comparing Total Bac^+^ to Total Bac^−^ cells, we found that macrophages with bacteria engulfed had significantly increased expression levels of genes that are involved in the inflammatory response through activation of TNF, INFγ and IFNα, and genes that are involved in the production of interleukins through the JAK–STAT signalling pathway, such as *IL1B*, *IL6* and *IL10*. Macrophages with bacteria engulfed also overexpressed the chemokines *CCL2*, *CCL4*, *CCL8*, *CCL7*, *CXCL1* and *CXCL10* (Extended Data Fig. 6k,l and Supplementary Table 7). This gene-expression signature was observed when analysing cells associated with bacteria in general (Extended Data Fig. 6k,l), but also when assessing specific bacterial genera, including *Fusobacterium* and *Treponema* (Fig. 3f,g and Supplementary Table 7). Furthermore, at the specimen level, the bacterial load from each OSCC specimen was positively correlated with the potent neutrophil chemoattractant *CXCL8* and negatively correlated with the expression of *CD3E* (Extended Data Fig. 7a), supporting the DSP findings that intratumoral bacteria-colonized microniches are immunosuppressive by recruiting neutrophils and excluding CD3^+^ T cells (Fig. 2 and Extended Data Fig 3c,d).

Unlike our findings in ‘macrophages with bacteria engulfed’ single cells, in which the response appears generalized to the presence of bacterial lipopolysaccharide or other widespread damage-associated molecular patterns, in epithelial single cells, specific dominant taxa such as *Fusobacterium* and *Treponema* enhanced signatures of cancer progression. Overall, this shows that the cell-associated members of the intratumoral microbiota can drive heterogeneity in patient tumours at the single-cell level within immune and epithelial populations.

**Extended Data Fig. 8:**
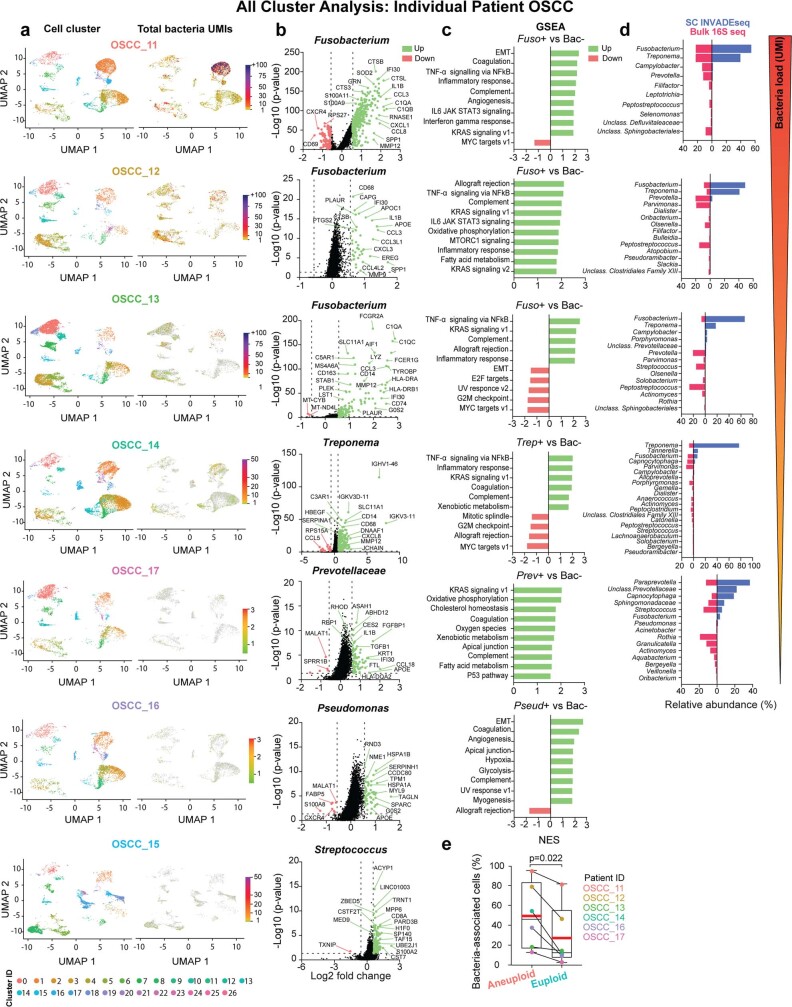
Differential gene expression, GSEA and microbiome analysis for each individual patient with OSCC. **a**, UMAP plots showing host cell annotation and detected bacterial transcripts (UMI) from each patient with OSSC as indicated. **b**, Volcano plots indicate the differential gene-expression profile comparing total bacteria-associated cells against total bacteria-negative cells in the entire sample for the dominant bacterial genera for each patient with OSCC; a ≥ 3 bacteria UMI cut-off was applied for samples OSCC_11, OSCC_12, OSCC_13 and OSCC_14. Dashed lines indicate the threshold of significant gene expression defined as the Log2 fold change ≥0.58 and ≤−0.58 with a -Log10 p value ≥1.301 following LMM analysis and Benjamini–Hochberg multiple-correction testing. **c**, GSEA analysis showing the top 10 signalling pathways that are differentially regulated comparing total bacteria-associated cells against total bacteria-negative cells in the entire sample for the respective dominant bacterial species for each patient with OSCC as indicated. A ≥ 3 bacteria UMI cut-off was applied for samples OSCC_11, OSCC_12, OSCC_13 and OSCC_14. No differentially regulated pathways were detected in OSCC_15 based on *Streptococcus* positive and negative cells. **d**, Microbiome analysis using the INVADEseq technique (SC INVADEseq) on single cells and bulk 16S rRNA gene sequencing (Bulk 16S seq) on tissue homogenate from the same dissociated tissue piece for 5 of the patients with OSCC. Genera with ≥ 1% relative abundance in either SC INVADEseq or Bulk 16S seq data are shown. The coloured bar (right) indicates the bacterial load (UMI count) in which the OSCC samples were arranged from the highest to lowest based on the bacterial UMI count **e**, Percentages of bacteria-associated cells in the aneuploid and euploid enriched epithelial cell clusters for each patient with OSCC (data points), 6 patients that contained both aneuploid and euploid epithelial cells are included. Box-whiskers indicate medians and the interquartile range. The box represents the middle 50% of scores for each group. Red bars indicate the mean of the combined data. p-values indicates statistical significance calculated by two-tailed paired t test.

An independent analysis of tumour single-cell data from the individual patients with OSCC revealed inter-patient heterogeneity in bacterial load, dominant cell-associated bacterial taxa and magnitude of the inflammatory gene-expression response (Extended Data Fig. 8a–d and Supplementary Tables 8–10). Similar to the integrated analysis, the percentage of bacteria-associated single cells is significantly higher in the aneuploid cancer epithelial cell cluster (cluster 3) compared to the euploid epithelial cell clusters (Extended Data Fig. 8e). This single-cell analysis of individual patients shows that specific cell-associated bacteria can significantly affect intratumoral heterogeneity at the single-cell level (Extended Data Fig. 8a–d and Supplementary Tables 8–10).

## Bacteria-induced migration of cancer cells

**Fig. 4:**
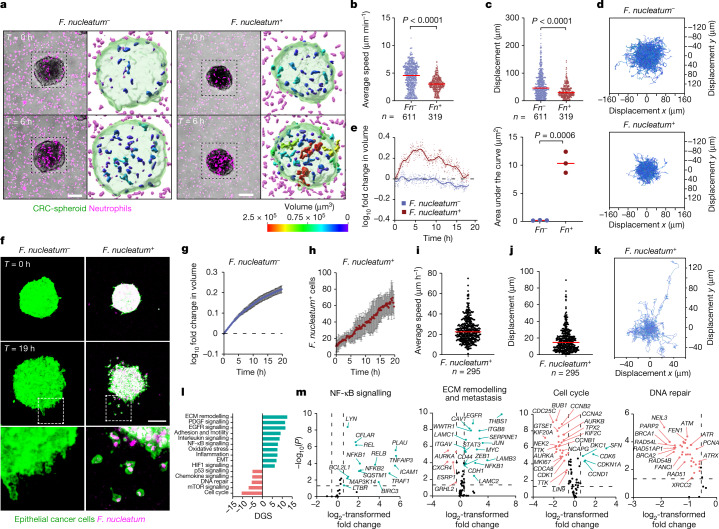
*F. nucleatum* induces neutrophil swarming and the migration of cancer epithelial cells. **a**, Live-cell confocal imaging showing neutrophil movements relating to CRC spheroids without (left) or with (right) *F.* *nucleatum*. Colour bars represent neutrophil cluster volume (µm^3^). Scale bars, 100 μm. **b**,**c**, Average speed (**b**) and cell displacement (**c**) of neutrophils migrating inside untreated control (lilac) and *F.* *nucleatum*-treated (red) spheroids. Red bars indicate mean. Data points represent individual tracks; *n* indicates the number of tracks per condition; three independent experiments. *P* values calculated by Mann–Whitney test. **d**, Neutrophil cell trajectory plots. **e**, Left, the log_10_-transformed fold change in volume over time of neutrophil clusters relative to the initial volume (*T* = 0 h). Data points represent average volume per time point, per condition. Right, quantification of the area under the curve for the fold change in volume. **f**, Confocal microscopy of HCT116 spheroid invasion capabilities without (left) or with (right) *F.* *nucleatum* over 19 h. Inset images represent differences in migration modes. Scale bar, 100 μm. **g**, The log_10-_transformed fold change in volume over time, representing the expansion rate of uninfected CRC spheroids. Error bars, s.d. **h**, Number of *F.* *nucleatum*-positive single cancer cells detaching from the spheroid over time. Error bars, s.d. **i**,**j**, Average speed (**i**) and cell displacement (**j**) of single cells escaping the *F.* *nucleatum*-infected spheroid. Red bars indicate mean. Data points represent individual tracks; *n* indicates the number of tracks per condition; three independent experiments. **k**, Cell trajectories of invading cancer cells escaping the *F.* *nucleatum*-infected spheroids. **l**, Signalling pathway analysis of CRC spheroids infected with *F. nucleatum* compared to uninfected control. The directed global significance (DGS) score was calculated as the square root of the mean squared *t*-statistic for genes in a gene set. ECM, extracellular matrix. **m**, Volcano plots of differential gene expression for selected pathways in *F.* *nucleatum*-infected spheroids compared to uninfected controls. Dashed lines indicate the threshold of significance, defined as log_2_-transformed fold change ≤ −0.58 and ≥ 0.58 and −log_10_(*P*) ≥ 1.301 after LMM analysis and Benjamini–Hochberg multiple-correction testing.

**Extended Data Fig. 9:**
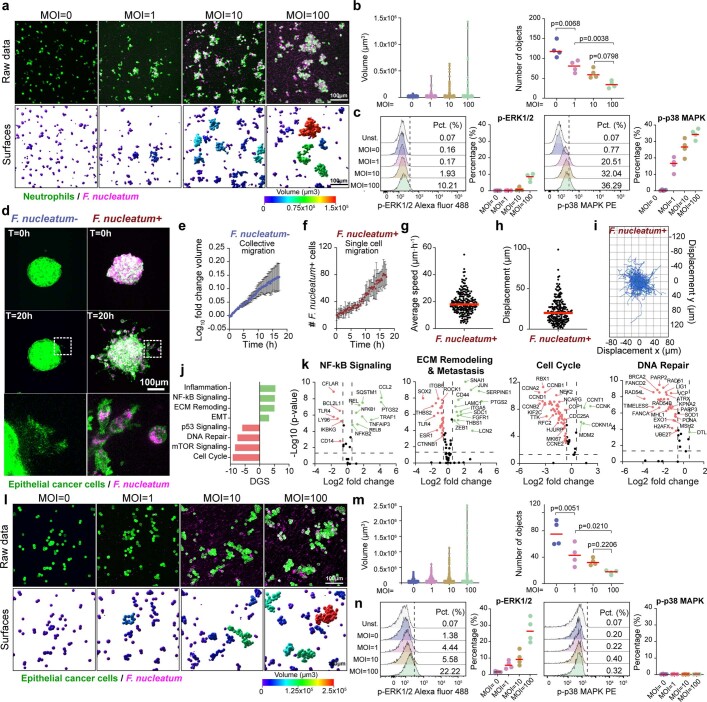
*F. nucleatum* induces the formation of cell clusters in immune and cancer cells. **a**, Confocal images showing cluster formation of differentiated neutrophils derived from human HL-60 cells (green) co-cultured with *F. nucleatum* (pink) at different multiplicity of infection (MOI). Top micrographs display the raw imaging data. Bottom micrographs display the corresponding mask surfaces for each experimental condition by using Imaris software. Colour bar indicates the size (volume µm^3^) of the objects. **b**, Left: Violin plot indicates the quantification of volume of individual neutrophil clusters (data points) in the present of *F. nucleatum* as shown in (a); combined data from 4 independent experiments. Right: Dot plot shows the number of neutrophil clusters (objects) per field of view as indicated in (a). Data points represent the number of cell objects for each independent experiment (n = 4). p-values were calculated by one-way ANOVA followed by Bonferroni multiple comparison test. **c**, Flow cytometry plots show the levels of phosphorylation of ERK and p38 MAPK in neutrophils treated with *F. nucleatum*. Corresponding dot plots indicate the level of phosphorylation for each independent experiment (data points; n = 4). **d**, Spheroids derived from a mouse CRC cell line CT26WT were treated with or without *F. nucleatum* for 12 h and then embedded in collage matrices. The cell invasion capabilities for both conditions were evaluated using live-cell confocal imaging over a period of 19 h. Amplified images show the difference in the migration mode from both conditions. **e**, Log_10_ fold change volume over time of uninfected CRC spheroids revealing the expansion rate of uninfected control spheroids. Data points indicate the average values from three independent experiments. Error bars indicate the standard deviation (SD). **f**, Number of *F. nucleatum*-positive cancer cells that detached from the spheroid mass as single motile cells from three independent experiments. Errors bars indicate the SD. **g-h**, Distribution of the average speed and cell displacements of single cells that escape the spheroid mass infected with *F. nucleatum*. Combined data from three independent experiments. Red bars indicate the mean. Data points represents individual tracks; “n” indicates number of tracks per condition. **i**, Cell trajectories from an origin point of invading cancer cells that escape the spheroids infected with *F. nucleatum*. **j**, Signalling pathway analysis of CRC spheroids infected with *F. nucleatum* in comparison to uninfected control spheroids. **k**, Volcano plots showing the regulation of genes in selected signalling pathways in spheroids infected with *F. nucleatum* in comparison to uninfected spheroids. Dashed lines indicate the threshold of significant gene expression defined as the Log_2_ fold change ≤−0.58 and ≥0.58 with a -Log_10_ p value ≥1.301 following LMM analysis and Benjamini–Hochberg multiple-correction testing. **l**, Confocal images showing cell cluster formation of cancer cells derived from the human cell line HCT116 treated with *F. nucleatum* at different MOI as it is indicated. **m**, Left: Violin plot indicates the quantification of volume of individual cancer cell clusters (data points) in the present of *F. nucleatum* as shown in (l); combined data from 4 independent experiments. Right: Dot plot shows the number of cancer cell objects per field of view as indicated in (l). Data points represent the number of clusters for each independent experiment (n = 4). p-values were calculated by one-way ANOVA followed by Bonferroni multiple comparison test. **n**, Flow cytometry plots show the levels of phosphorylation of ERK and p38 MAPK in cancer cells treated with *F. nucleatum*. Corresponding dot plots indicate the level of phosphorylation for each independent experiment (data points; n = 4).

To evaluate the direct interactions of a dominant member of the intratumoral microbiota with immune or epithelial cancer cells, we used a reductionist in vitro co-culture approach. We co-cultured CRC epithelial spheroids with an *F.* *nucleatum* CRC isolate, followed by embedding in collagen matrices that contained neutrophils distributed uniformly throughout the gel ( Supplementary Methods). By using live-cell confocal microscopy, the embedded neutrophils are tracked inside *F.* *nucleatum*-infected spheroids and could be compared to control uninfected spheroids (Fig. 4a and Supplementary Video 1). In the absence of *F.* *nucleatum*, neutrophils migrated freely inside the spheroids with an average speed of 4.329 µm min^−1^ ± 0.08766 (s.e.m.) (Fig. 4b) and average cell displacement of 57.21 µm (Fig. 4c,d). In the presence of *F.*  *nucleatum*, neutrophils responded to bacterial infection by reducing their migration capabilities with an average speed of 3.593 µm min^−1^ ± 0.08561 (s.e.m.) (Fig. 4b) and a mean cell displacement of 34.53 µm (Fig. 4c,d) as they formed cell clusters inside the spheroids, reaching a maximum size at around 6 h, after which the clusters gradually started to disassemble^24^ (Fig. 4e). The recruitment and retention of neutrophils to the cancer cell spheroids that were infected with *F.* *nucleatum* indicates that the intratumoral microbiota has an active role in the enrichment of neutrophils within bacteria-colonized microniches of patient tumours, as was observed through spatial profiling (Fig. 2b). Neutrophil cluster formation (Extended Data Fig. 9a,b) was accompanied by significantly increased levels of phosphorylation of ERK and p38 MAPK in response to *F.* *nucleatum* (Extended Data Fig. 9c). This suggests that the observed upregulation of phosphorylation of ERK and p38 MAPK in bacteria-colonized microniches within patient tumours is in part driven by a myeloid response to intratumoral bacteria (Fig. 2b).

We also show that the CRC epithelial cells infected with *F.* *nucleatum* detached from the spheroid mass and migrated into the surrounding collagen gel as single epithelial cells (Fig. 4f and Supplementary Video 2). By contrast, uninfected cancer epithelial cells invaded as a collective, as the entire spheroid mass spread through the gel at an average expansion rate of 1.34 × 10^5^ µm^3^ h^−1^ (Fig. 4g). Notably, intracellular bacteria were detected in migratory cancer cells as they invaded the collagen gel (Fig. 4h). Cell-tracking analysis showed that invading cancer cells migrated with an average speed of 19.99 µm h^−1^ ± 0.827 (s.e.m.) (Fig. 4i) and a mean displacement of 20.54 µm (Fig. 4j, k). Similar findings were observed when assessing a mouse CRC cell line, in which *F.* *nucleatum*-infected cancer cells invaded the surrounding collagen gel as individual cells (Extended Data Fig. 9d–i and Supplementary Video 3). Invasive bacteria not only promote the invasion of cancer cells in collagen matrices^25^, but also change the motility patterns of infected cancer cells and thereby promote cell heterogeneity at a functional level, as shown by the broad range of cell-displacement and velocity values measured in cells as they migrate through the gel (Fig. 4i–k).

To identify the altered signalling pathways involved, spheroids containing *F.* *nucleatum* were dissociated for transcriptomic analysis using the nCounter platform. Differential expression at the pathway level indicated that exposure to *F.* *nucleatum* led to a significant upregulation of signalling pathways that are involved in cancer progression, including remodelling of the extracellular matrix, metastasis and cell adhesion and migration, as well as an upregulation of signalling through growth factors such as EGFR and PDGF, and signalling through the EMT and NF-κB pathways (Fig. 4l,m). Furthermore, *F.* *nucleatum*-treated spheroids exhibited a downregulation of signalling pathways related to the cell cycle, DNA damage repair and p53 signalling (Fig. 4l,m and Supplementary Table 11). Transcriptional modifications induced by *F.* *nucleatum* were also found in the mouse CRC cell line, with an upregulation of metastasis- and EMT-related genes (Extended Data Fig. 9j,k and Supplementary Table 11). Moreover, we observed a significant downregulation in *MKI67* expression in *F.* *nucleatum*-exposed cancer spheroids (Supplementary Table 11), consistent with the downregulation of Ki-67 in bacteria-colonized microniches that was observed in the DSP analysis from patient tumours (Fig. 2) and the negative correlation with bacterial load that was observed in the whole-sample analysis from the scRNA-seq data (Extended Data Fig. 7a). Of note, despite lower proliferation levels, the spheroids infected with *F.* *nucleatum* had increased single-cell migration capabilities (Fig. 4f and Supplementary Video 2). This is of particular interest given our previous finding that the dominant intratumoral microbiota—including *F.* *nucleatum*—persists with CRC in distant-site metastases^8^, and warrants further investigation into the effect of the intratumoral microbiota on the cell migration–proliferation dichotomy in cancer^26,27^. Finally, flow cytometry analysis showed that cancer epithelial cells also upregulated the levels of phosphorylation of ERK1 and ERK2 in the presence of *F.* *nucleatum* during the formation of cancer cell clusters^28^ (Extended Data Fig. 9l–n). Together, these data show that *F.* *nucleatum* derived from human CRC actively induces the recruitment of myeloid cells at the sites of bacterial infection and promotes transcriptional changes in CRC epithelial cells that facilitate invasion to the surrounding environment and may confer quiescent properties.

## Discussion

Historically, tumour heterogeneity was attributed solely to intrinsic genetic alterations in cancer cells during clonal expansion^29^. Studies in the 1990s^30,31^ revealed that extrinsic factors derived from the TME^32,33^ have an important role in tumorigenesis. The intercellular interactions between cancer cells and other non-malignant cell populations such as fibroblasts, endothelial and immune cells in the TME are known to contribute to tumour heterogeneity by promoting transcriptomic changes in transformed cells as the cancer evolves^34–36^. As our understanding of the TME advances, so too does our understanding of what affects tumour heterogeneity. Genomics-based studies have shown that most major types of human cancer contain an intratumoral microbiota^2,16^. These microbial communities vary by cancer type, and specific bacteria can contribute to the initiation and progression of cancer, affect the response of patients to treatment and thus affect survival^2,8,12,14–16,21,37^. Nevertheless, the intrinsic heterogeneity present has made it difficult to understand the interplay between different components of the TME, including bacteria–host interactions within the native tissue context. The development of spatial transcriptomics^38^ and scRNA-seq technologies^39,40^ has enabled eukaryotic components of the TME to be studied, but the effect of the intratumoral microbiota in the TME has so far been overlooked. In this study, by adapting and applying these technologies, we conclude that the intratumoral microbiota is heterogeneously distributed across human tumours. Further, we show that it is a fundamental component of the TME that can alter the biology of distinct cellular compartments, affecting anti-tumour immunity and the migration of cancer epithelial cells. By activating transcriptional factors from the JUN and FOS family, intracellular bacteria can generate gene signatures that are consistent with cancer cell invasion, metastasis, DNA damage repair and cell dormancy. Likewise, invasive bacteria are responsible for recruiting myeloid cells to induce an inflammatory response through JAK–STAT signalling, promoting T cell exclusion and tumour growth by secreting specific interleukins and chemokines into the surrounding environment. Although we focused here on two cancer types at the extremes of the gastrointestinal tract, the tools and technologies that we describe could be applied to analyse the 33 major cancer types that have so far been shown to contain an intratumoral microbiota. Analyses that move beyond correlative associations of the microbiota with human cancers, towards those that assess the functional effect of the intratumoral microbiota, will identify molecular and cellular targets for the prevention and treatment of such cancers. Collectively, this work shows that the distribution of the intratumoral microbiota within patient tumours is not random, but rather, that the microbiota is highly organized in microniches with immune and epithelial cell functions that support cancer progression.

### Reporting summary

Further information on research design is available in the Nature Portfolio Reporting Summary linked to this article.

## Online content

Any methods, additional references, Nature Research reporting summaries, source data, extended data, supplementary information, acknowledgements, peer review information; details of author contributions and competing interests; and statements of data and code availability are available at 10.1038/s41586-022-05435-0.

### Supplementary information

Supplementary Information This file contains Supplementary Fig. 1, descriptions for Supplementary Videos 1–3 and Supplementary Tables 1–11 (files supplied separately), and Supplementary Methods.

Reporting Summary

Supplementary Tables Supplementary Tables 1–11; see Supplementary Information document for full descriptions.

Video 1 | **Neutrophils formed clusters inside CRC spheroid infected with** ***F. nucleatum***. Live-cell confocal imaging, over 18 h, of neutrophils (magenta) embedded in collagen matrices migrating in relation to CRC spheroids previously infected with or without *F. nucleatum* at MOI = 100 for 12 h. Magnified field of views demonstrate the cell surfaces of migrating neutrophils inside the boundaries of the LifeactGFP fluorescence (green) from CRC spheroids for both conditions. Colour bars indicate the volume (μm3) of the objects that are generated inside the CRC spheroids. Scale bar, 100 μm. Time in h:min:s.

Video 2 | ***F. nucleatum*** **changes the invasion properties of human CRC cells**. Live-cell confocal imaging, over 18 h, of human CRC spheroids expressing the LifeactGFP construct (green) previously treated with or without *F. nucleatum* at MOI = 100 for 12 h. Bacteria were labelled with 5 μg/mL FM 4-64 FX membrane dye (magenta). Magnified field of views indicate the difference in cell migration capabilities between conditions. Control CRC spheroids invade the collagen matrices as a collective whereas in *F. nucleatum*-infected CRC spheroids, cancer cells invade the surrounding as single migrating cells. Scale bar, 100 μm. Time in h:min:s.

Video 3 | F. nucleatum changes the invasion properties of mouse CRC cells. Live-cell confocal imaging, over 18 h, of mouse CRC spheroids expressing the LifeactGFP construct (green) previously treated with or without *F. nucleatum* at MOI = 100 for 12 h. Bacteria were labelled with 5 μg/mL FM 4-64 FX membrane dye (magenta). Magnified field of views indicate the difference in cell migration capabilities between conditions. Control CRC spheroids invade the collagen matrices as a collective whereas in *F. nucleatum*-infected CRC spheroids, cancer cells invade the surrounding as single migrating cells. Scale bar, 100 μm. Time in h:min:s.

## Data Availability

Raw sequencing data from bulk 16S ribosomal RNA gene sequencing, 10x Visium spatial transcriptomics and INVADEseq bacterial 16S rRNA and human (GEX) gene libraries are available in the NCBI Sequence Read Archive (SRA) repository under the Bioproject accession number PRJNA811533. PathSeq, Cell Ranger and Space Ranger analyses used GRCh38 as the human genome reference.

## Code availability

Custom code for data processing and analysis of 10x Visium spatial transcriptomics and scRNA-seq data is available at https://github.com/FredHutch/Galeano-Nino-Bullman-Intratumoral-Microbiota_2022.
