# Supplementary material for: Effect of the intratumoral microbiota on spatial and cellular heterogeneity in cancer
Source: Nature. 2022 Nov 16;611(7937):810–7. doi: 10.1038/s41586-022-05435-0 (PMC9684076; doi:10.1038/s41586-022-05435-0)
Supplement: Supplementary file 1 — This file contains Supplementary Fig. 1, descriptions for Supplementary Videos 1–3 and Supplementary Tables 1–11 (files supplied separately), and Supplementary Methods. [file 41586_2022_5435_MOESM1_ESM.pdf]

nature portfolio

https://doi.org/10.1038/s41586-022-05435-0

Supplementary information

Effect of the intratumoral microbiota on spatial and cellular heterogeneity in cancer

In the format provided by the authors and unedited

Supplementary Information Guide

Impact of Intratumoral Microbiota on Spatial and Cellular Heterogeneity in Cancer

Jorge Luis Galeano Niño[1], Hanrui Wu[1,¥], Kaitlyn D. LaCourse[1,¥], Andrew G. Kempchinsky[1], Alexander Baryiames[1], Brittany Barber[2], Neal Futran[2], Jeffrey Houlton[2], Cassie Sather[3], Ewa Sicinska[4] , Alison Taylor[5], Samuel S. Minot[6], Christopher D. Johnston[7]* , Susan Bullman[1]*.

Supplementary Information Guide

Table of content

- **Supplementary Figure 1 : page 1**
- **Supplementary Video Legends: page 2**
- **Supplementary Table Legends: pages 3-4**

Supplementary File contains:

- **Methods**
- **Supplementary Figure 1**
- **Supplementary Videos 1-3**
- **Supplementary Tables 1-11**

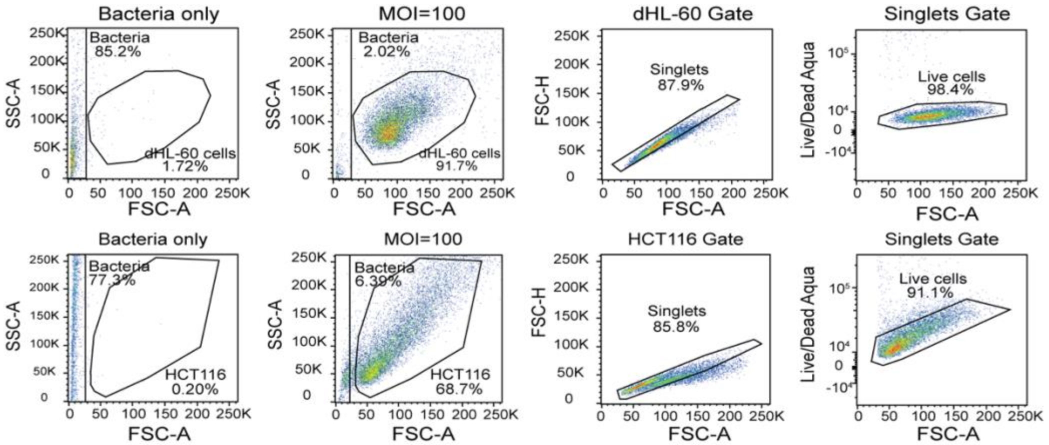

Supplementary Figure 1| Flow Cytometry Gate Strategy

Flow cytometry plots showing the gate strategy for the fluorescent histograms described in Extended Data Fig. 9c for dHL-60 cells (Upper panels) and **Extended Data Fig. 9n** for HCT116 cells (Bottom panels). A sample containing only bacteria was processed to gate out undesirable bacteria cell events. From the MOI=100 condition as a representative example, the doublets events were excluded from the analysis by plotting the forward scatter height (FSC-H) vs. forward scatter area (FSC-A) density plot. By selecting the singlet gate, the live cells were identified by gating the negative Live/Dead aqua unstained cells. The fluorescent histograms from **Extended Data Fig. 9c and 9n** demonstrate the phosphoprotein levels of p38 MAPK (Thr180/Tyr182) and p44/42 MAPK (Erk1/2) (Thr202/Tyr204) in dHL-60 and HCT116 cells by selecting the viable cells from the singlet gate. An unstained control was run to determine the percentages of the fluorescent signal for each kinase.

Supplementary Video Legends

Supplementary video 1| Neutrophils formed clusters inside CRC spheroid infected with *F. nucleatum*. Live-cell confocal imaging of neutrophils (magenta) embedded in collagen matrices migrating in relation to CRC spheroids previously infected with or without *F. nucleatum* at MOI=100 for 12h. Magnified field of views demonstrate the cell surfaces of migrating neutrophils inside the boundaries of the LifeactGFP fluorescence (green) from CRC spheroids for both conditions. Color bars indicate the volume (μm^3) of the objects that are generated inside the CRC spheroids. Scale bar, 100 µm. Time in h:min:s.

Supplementary video 2| *F. nucleatum* changes the invasion properties of human CRC cells. Live-cell confocal imaging of human CRC spheroids expressing the LifeactGFP construct (green) previously treated with or without *F. nucleatum* at MOI=100 for 12h. Bacteria were labelled with 5µg/mL FM 4-64 FX membrane dye (magenta). Magnified field of views indicate the difference in cell migration capabilities between conditions. Control CRC spheroids invade the collagen matrices as a collective whereas in *F. nucleatum*-infected CRC spheroids, cancer cells invade the surrounding as single migrating cells. Scale bar, 100 µm. Time in h:min:s.

Supplementary video 3| *F. nucleatum* changes the invasion properties of mouse CRC cells. Live-cell confocal imaging of mouse CRC spheroids expressing the LifeactGFP construct (green) previously treated with or without *F. nucleatum* at MOI=100 for 12h. Bacteria were labelled with 5µg/mL FM 4-64 FX membrane dye (magenta). Magnified field of views indicate the difference in cell migration capabilities between conditions. Control CRC spheroids invade the collagen matrices as a collective whereas in *F. nucleatum*-infected CRC spheroids, cancer cells invade the surrounding as single migrating cells. Scale bar, 100 µm. Time in h:min:s**.**

Supplementary Table Legends

Supplementary Table 1: Patient Data Table
Colorectal cancer (CRC) and Oral cavity squamous cell carcinoma (OSCC) primary tumor samples: The table indicates the list of human specimens used for this study including information related to the specimen and tissue type and the platform that was implemented to generate the corresponding data. Note: There are multiple tumor specimen aliquots from an individual patient tumor. Each specimen aliquot from an individual tumor has a unique Specimen ID but an identical Patient ID.

Supplementary Table 2: Bacterial 16S rRNA gene sequencing from CRC tumor samples.
Table indicates the bacteria composition at genus level for each piece of tumor tissue denoted as A, B, C and D from a total of 11 CRC samples. DNA was extracted for each tumor tissue for bacterial 16S ribosomal RNA gene sequencing. Taxonomical annotations were performed using the Uclust from Qiime v.1.9.1 with the Zymo Research Database.

Supplementary Table 3: Bacterial genera detection, UMI and read counts from 10x Visium spatial transcriptomics.
The table indicates the list of bacterial taxa at the genus level that were detected in the tumor tissue from an oral cavity squamous cell carcinoma (OSCC) and colorectal cancer (CRC) patient tumor, respectively. Captured bacterial transcripts were released from the capture spots on the 10x Visium slide and sequence using Illumina NextSeq 2000 or NovaSeq 6000 system platform. Reads that did not map to the human genome or transcriptome (unmapped reads), were then aligned against microbial databases through GATK PathSeq software providing microbiome composition for each sample. The number of capture spots positive for each taxa and the number of reads and UMIs, along with percent relative abundance (by UMI count) are shown below.

Supplementary Table 4: GeoMX-DSP protein expression profiles.
Tables indicate the protein expression profile that are differentially expressed using the GeoMX-DSP platform comparing bacteria-positive areas of interest (AOI+) vs bacteria-negative areas of interest (AOI-) measuring a total of 77 protein targets. The analysis was performed in the immune (CD45+), epithelial (PanCK+) or combined (All) segments from oral cavity squamous cell carcinoma (OSCC) and colorectal cancer (CRC) tissue samples embedded in FFPE. Analysis at the microniche level denotes that the AOIs+ were compare against AOIs- from the same tissue samples, whereas at the tumor level analysis the AOIs were compared between tumors that were positive for bacteria against tumors that did not contain detectable bacteria. Data was normalized relative to housekeeping protein expression. p-values and fold change were calculated by applying a linear mixed effect model followed by Benjamini-Hochberg (BH) multiple correction test.

Supplementary Table 5: Single cell RNA sequencing; gene expression profile in cell lines. Integrated data.
Differential expression analysis between cellular groups was performed using the FindMarker function from the Seurat package based on the Wilcoxon Rank Sum test. The log2 fold change is calculated based on the difference in the gene expression average between two groups. Adjusted p-values were calculated using the Bonferroni correction test for each gene in the dataset. p-value <0.05 indicates significant differential gene regulation between experimental conditions.

Supplementary Table 6: Single-cell RNA sequencing; gene expression profile in the epithelial cell clusters from human OSCC tumors. Integrated data.
Differential expression analysis between cellular groups was performed using the FindMarker function from the Seurat package based on the Wilcoxon Rank Sum test. The log2 fold change is calculated based on the difference in the gene expression average between two groups. Adjusted p-values were calculated using the Bonferroni correction test for each gene in the dataset. p-value <0.05 indicates significant differential gene regulation between experimental conditions.

Supplementary Table 7: Single-cell RNA sequencing; gene expression profile in the monocyte-derived macrophage v1 cell cluster from human OSCC tumors. Integrated data.
Differential expression analysis between cellular groups was performed using the FindMarker function from the Seurat package based on the Wilcoxon Rank Sum test. The log2 fold change is calculated based on the difference in the gene expression average between two groups. Adjusted p-values were calculated using the Bonferroni correction test for each gene in the dataset. p-value <0.05 indicates significant differential gene regulation between experimental conditions.

Supplementary Table 8: Single-cell RNA sequencing; gene expression profile for each individual OSCC patient. All cluster analysis.
Differential expression analysis between cellular groups was performed using the FindMarker function from the Seurat package based on the Wilcoxon Rank Sum test. The log2 fold change is calculated based on the difference in the gene expression average between two groups. Adjusted p-values were calculated using the Bonferroni correction test for each gene in the dataset. p-value <0.05 indicates significant differential gene regulation between experimental conditions.

Supplementary Table 9: Single-cell RNA sequencing; Gene Set Enrichment Analysis (GSEA) for each individual OSCC patient. All cluster analysis.
Gene Set Enrichment Analysis (GSEA) between cellular groups was performed using the FindMarker function from the Seurat package based on the Wilcoxon Rank Sum test. The log2 fold change is calculated based on the difference in the gene expression average between two groups. Adjusted p-values were calculated using the Bonferroni correction test for each gene in the dataset. p-value <0.05 indicates significant differential gene regulation between experimental conditions.

Supplementary Table 10: Single-cell RNA sequencing; Microbiome composition, number of associated single cells and UMI and read counts for each individual OSCC patient.
Table indicates the microbiome composition at the genus level for each OSCC tumor including the number of bacteria-positive single cells, number of UMI transcripts and total number of reads for each taxa identified.

Supplementary Table 11: nCounter gene expression profile.
Tables indicate the gene expression profile that are differentially expressed using the nCounter platform comparing CRC spheroids infected with or without *F. nucleatum* at MOI=100 for 12h. Data was normalized relative to housekeeping gene expression from the code set content. P-values and fold change were calculated by applying a linear mixed effect model. FDR p-value adjustment was performed with the Benjamini–Yekutieli (BY) method.

METHODS

Patient specimens: All patients included in the analysis were diagnosed with either colorectal adenocarcinoma or oral cavity squamous cell carcinoma and were treatment naive at the time of tumor surgical resection. Patients signed an informed consent for the collection and analysis of their tumor specimens. The use of patient specimens for this work was approved by the Fred Hutchinson Cancer Center Institutional Review Board (IRB) under the following protocol numbers RG #: 1006552, 1006974. As noted in **Supplementary Table 1**, for several patients we obtained multiple tumor specimen aliquots from an individual patient primary tumor. These tumor specimen aliquots are represented by unique specimen identification numbers but an identical patient identification number. Each individual patient had a single tumor (primary tumor) assessed. Patient specimens included formalin fixed paraffin embedded tissue, OCT embedded tissue, fresh frozen tissue and fresh tissue.

Cell line culture conditions:
HL-60 cell lines were grown in medium RPMI 1640 supplemented with 10% FBS, 10 mM HEPES, 10 mM Sodium pyruvate, 100 U/ml penicillin and 100µg/ml streptomycin (Pen/Strep), 2mM L-glutamine and 50 µM β2-mercaptoethanol (all from Gibco, ThermoFisher Scientific, Waltham, MA, USA). Prior to imaging, HL-60 cells were differentiated by treatment with 1.3% DMSO for 5 days. All human colon cancer epithelial cells lines (HT-29 and HCT 116) were cultured in McCoys 5A with L-glutamine (Corning), Pen/Strep and supplemented with 10% (v/v) fetal bovine serum (Sigma). Pen/Strep was not used in cell line co-culture experiments with bacteria. Cell cultures were incubated at 37°C in 5% CO^2. Cell lines were not authenticated. Cell lines were routinely screened for mycoplasma contamination through the Specimen Processing & Research Cell Bank facility at the Fred Hutch using the MycoProbe kit (R & D Systems, Minneapolis, MN, USA). All cell lines included in this study tested negative for mycoplasma immediately prior to each experiment.

Bacteria culture conditions:
All bacterial strains were grown from cryostocks on fastidious anaerobe agar plates (FAA; Grainger, Neogen, Lansing MI, USA) supplemented with 10% defibrinated horse blood (Lampire Biological Laboratories, Fisher, Pipersville PA, USA). Bacterial culturing occurred under anerobic conditions in an anaerobic chamber (Anaerobe Systems AS-580) and incubated at 37°C. The taxonomic identity of pure cultures was confirmed by colony PCR targeting the 16S rRNA (342F and 1492R) and BLASTn analysis following sanger sequencing as described previously[8].

16S rRNA gene amplicon sequencing of tumor tissue:
Individual fresh-frozen CRC samples (n=11; **Extended Data Fig. 1a-d**) were split in 4 pieces and stored at -20°C in 1.5 mL tubes and shipped on dry ice in insulated boxes to Zymo Research (Zymo Research, Irvine, CA) for bacterial 16S ribosomal RNA gene sequencing. DNA extraction was performed by using the Quick-16S™ NGS Library Prep Kit (Zymo Research). Bacterial 16S ribosomal RNA sequencing was performed by Zymo Research. Briefly, libraries were prepared using the Quick-16 NGS Library Prep Kit (Zymo Research). Predesigned primers amplified the conserved V3-V4 region of the 16S rRNA gene. The sequencing library was prepared by RT-PCR, cleaned through Select-a-Size DNA Clean & Concentrator kit (Zymo Research) and then quantified with TapeStation (Agilent Technologies, Santa Clara, CA) and Qubit (Thermo Fisher Scientific, Waltham, WA). The DNA library was sequence on Illumina® MiSeq platform (lllumina, Inc., San Diego, CA, USA). Taxonomic annotations were performed using the Uclust from Qiime v.1.9.1 with the Zymo Research Database. Composition visualization, Principal Component Analysis (PCA) of beta-diversity (Bray-Curtis Index) with PERMANOVA and Dendrogram analysis (beta-diversity with Ward clustering)

were performed using MicrobiomeAnalyst software[41]. For the OSCC patients involved in the INVADEseq method (**Extended Data Fig 8**), an aliquot of tissue homogenate following tissue dissociation with the OctoMACS instrument (described in more detail below) was stored at -80. This tissue homogenate was processed for bacterial 16S rRNA gene sequencing (n=5 OSCC cases; **Extended Data Fig 8**) via the same procedure as above.

RNAscope-FISH:

RNAscope multiplex fluorescent reagent 2.5HD kit assay (Advanced cell diagnostics, Newark, CA, USA) was performed to visualize the distribution of bacterial communities including *F. nucleatum* along the tumor tissue. For formalin-fixed paraffin embedded (FFPE) tissue were cut into 5 µm sections using the Leica RM2255 microtome (Leica Microsystem), mounted onto superfrost plus slides and air dried overnight at room temperature. Slides were baked for 1h at 60°C using the HybEZ™ II hybridization system oven (Advanced cell diagnostics). Then the slides were deparaffinized using several cycles of xylene and 100% ethanol washes. Endogenous peroxidases were blocked using a hydrogen peroxide solution for 10 min at room temperature. Antigen target retrieval was performed using an Oster Steamer where the slides were placed in a tissue staining dish filled with 200 ml of RNAscope 1X target retrieval reagent inside the steam bowl for 15min at 99°C. Temperature was monitored by inserting a digital thermometer inside the steamer. Then the slides were washed with 100% ethanol for 3 min, air dried and a hydrophobic barrier was drawn around the tumor tissue. Slides were treated with RNAscope protease plus for 20 minutes at 40°C using the HybEZ™ II oven. For samples embedded in cryo-embedding medium (OCT), the blocks were equilibrated at -20°C in the Leica CM 1850 UV cryostat (Leica Microsystem) for 1h and 13 µm thick sections were cut and mounted onto superfrost plus slides. The sections were dried for 60 min at -20°C to retain tissue adherence. The sections were then fixed with 10% neutral buffered formalin (NBF) for 45 min at room temperature and dehydrated by several ethanol wash cycles. Slides were air dried, and a hydrophobic barrier were drawn around the tissue sample. Samples were treated with hydrogen peroxide for 10 min and treated with RNAscope Protease IV for 30 minutes at room temperature. For FFPE and OCT samples, each slide was treated with 150 µl of a RNAscope probe mix against *F. nucleatum* (B-Fusobacterium-23S-3zz-C1; Advanced cell diagnostics) and with a Eubacteria probe (EB-16S-rRNA-C2; Advanced cell diagnostics) that targets a conserved 16S-rRNA region, thus detecting the bacterial communities that were present in the tumor tissue. As a negative control, the corresponding sequential slides were incubated with a probe that target an unrelated gene (DapB; accession # EF191515). The probes hybridization process was performed using the HybEZ™ II oven for 2h at 40°C. Following three amplification cycles, each probe channel was developed separately staining the *F. nucleatum*-C1 channel with 200 µl of Opal-570 and Eubacteria-C2 channel with 200µl of Opal-690 for 30 min at 40°C using the HybEZ™ II oven. Between each amplification and staining step, slides were washed twice in 1X RNAscope wash buffer for two minutes. Then, the slides were incubated with DAPI (ThermoFisher Scientific) for 30 sec at room temperature and placed them with ProLong Gold antifade mounting solution (ThermoFisher Scientific) prior imaging. RNAscope-FISH slides were scanned using Akoya Vectra Polaris (Akoya Biosciences, Marlborough, MA, USA) using a 40x objective. Images were acquired using tile scanning and Opal dyes were exposed with LED fluorescent light source using different filters to excite each fluorophore as followed: Opal 570 at 550nm excitation peak with 3.96ms exposure time, Opal 690 at 675nm excitation peak with 8.5ms exposure time and DAPI at 370nm excitation peak with 3.83ms exposure time.

RNAscope-CISH:

Chromogenic RNAscope 2.5 HD Duplex Assay (Advanced Cell Diagnostics) was performed to visualize tissue architecture and bacterial communities, including *F. nucleatum* for the selection of

regions of interest for GeoMx Platform. FFPE tissue were sectioned to 4um using the Leica RM2255 microtome (Leica Microsystem), mounted onto superfrost plus slides and air dried overnight at room temperature. Slides were baked for 1h at 60°C using the HybEZ™ II hybridization system oven (Advanced cell diagnostics). Deparaffination, peroxidase blocking, and antigen retrieval were performed as described above (see RNAscope-FISH). Slides were treated with RNAscope Protease Plus for 20 minutes at 40°C using the HybEZ™ II oven followed by adding 150µL of probe mixture containing EB-16S-rRNA-C1 and Fusobacterium-23S-3zz-C2 on the slides. Hybridization was performed for 2 hours in HybEZ™ II hybridization oven at 40°C. After six amplification steps, C2, was developed with alkaline phosphatase (AP) Enzyme and Fast Red. After four subsequent amplification steps, C1 was developed using horseradish peroxidase (HRP) enzyme and Green Substrate. Between each amplification and development step, slides were washed twice in 1X RNAscope wash buffer for two minutes. Slides were counterstained with 50% Gill's Hematoxylin for 30 seconds and then immediately rinsed in tap water for 30 seconds. Slides were then dipped in 0.02% ammonia water to result in blue hematoxylin staining. Slides were dried in hybridization over at 60°C for 15-30 minutes. Slides were then dipped in xylene and immediately mounted with Cytoseal prior to scanning. RNAscope-CISH slides were scanned using Akoya Vectra Polaris (Akoya Biosciences, Marlborough, MA, USA) using tile scanning and a 40x objective. Images were acquired using the bright field.

Macrodissection of Tumor Tissue Based on RNAscope *F. nucleatum* Positivity: Validation of RNAscope Approach:

As described earlier, RNAscope CISH using the *F. nucleatum* RNAscope probe was performed on 4um tissue sections. Regions of relatively high and low *F. nucleatum* positivity were marked by a pathologist. These regional markings were then transferred to unstained formalin fixed paraffin embedded (FFPE) tissue slides (two 20µm sections in diameter) were marked. Following tissue marking, a sterile disposable blade was used to scrape off the *F. nucleatum* relatively low regions of tissue first and these were collected in a sterile 2ml tube. Using a new sterile blade, the *F. nucleatum* relatively high regions of tissue were then scraped off the slide and collected in a separate sterile 2ml tube. *F. nucleatum* relatively high and low tissue regions (based on RNAscope staining) were then processed for DNA extraction as an input for *Fusobacterium*-specific quantitative PCR and microbiome analysis.

Quantitative PCR (qPCR):

A custom TaqMan primer/probe set was used to amplify *Fusobacterium* species DNA (Integrated DNA technologies, CA) as previously described[42]. The cycle threshold (Ct) values for *Fusobacterium* species were normalized to the amounts of human genomic DNA in each reaction by using a primer and probe set for the prostaglandin transporter (*PGT)* reference gene, and the fold difference ($2^{-\Delta Ct}$) in *Fusobacterium* load in tumor tissue was calculated as described before[43]. Each reaction contained 50ng of genomic DNA and was assayed in triplicate in 20 µL reactions containing 1× final concentration TaqMan Universal Master Mix (Applied Biosystems) and each TaqMan Gene Expression Assay (Applied Biosystems), in a 96-well optical PCR plate. Amplification and detection of DNA was performed with the ABI 7300 Real-Time PCR System (Applied Biosystems) using the following reaction conditions: 10 min at 95°C and 42 cycles of 15 s at 95°C and 1 min at 60°C. Cycle thresholding was calculated using the automated settings (Applied Biosystems). The primer and probe sequences for each TaqMan Gene Expression Assay were as follows: *Fusobacterium* species forward primer, 5'-AAGCGCGTCTAGGTGGTTATGT-3'; *Fusobacterium* species reverse primer, 5'-TGTAGTTCCGCTTACCTCTCCAG-3'; *Fusobacterium* species FAM probe, 5'-

CAACGCAATACAGAGTTGAG-3'. PGT forward primer, 5'-ATCCCCAAAGCACCTGGTTT-3'; PGT reverse primer, 5'-AGAGGCCAAGAT AGTCCTGGTAA-3'; PGT FAM probe, 5'-CCATCCATGTCCTCATCTC-3'.

10x Visium spatial transcriptomics:
CRC and OSCC samples were processed into OCT blocks as describe above. 13 µm frozen tissue section was cut and affixed to a Visium Spatial Gene Expression library preparation slide (10X Genomics, Pleasanton, CA, USA) containing 6.5mm x 6.5mm capture areas with 5000 oligo-barcoded spots. In some cases, large pieces of tissue were trimmed using the Leica CM1850 UV cryotome (Leica Microsystem) to fit the capture area. RNAscope images were used to allocate pieces of tissue that were positive for Eubacteria or *F. nucleatum* on the capture areas. The tissue samples were fixed in methanol and stained with hematoxylin-eosin reagents (Sigma-Aldrich). Images of hematoxylin-eosin-stained samples were acquired using a 10x objective from the Leica DMi8 microscope (Leica Microsystems). Permeabilization times and reverse transcription (RT) reaction length was determined using the Visium Spatial Tissue Optimization Kit (10X genomics). For the CRC sample the permeabilization time was 24 min and for the OSCC sample the permeabilization time was 12 min. Following permeabilization, RT reaction was performed at 53ºC for 45 min and second strand synthesis was subsequently generated on the 10x Visium expression slide using a thermocycler slide adaptor for the C1000 Touch Thermal Cycler (Bio-Rad laboratories, Hercules, CA, USA). Second strand synthesis was performed and then the cDNA was denatured by adding 35 µl 0.08 M KOH in each capture area well for 10 min at room temperature and then transferred to tubes for cDNA amplification by PCR and cleanup by using SPRIselect beads (Beckman Coulter). Final libraries were generated by enzymatic fragmentation, size selection, End Repair, A-tailing, Adapter ligation, and PCR. Library quality was evaluated using Agilent 4200 TapeStation (Agilent Technologies) and subsequent sequence using the Illumina NextSeq 2000 or NovaSeq 6000 system (lllumina, Inc).

Bioinformatic analysis of 10x Visium spatial transcriptomic data:
The Visium expression matrix for each sample was processed using Seurat v4.0.4 (Satija Lab, New York, NY, USA)[44]. Visium spots with more than three transcripts (UMIs) were defined as valid spots and processed for downstream analysis. Normalization and variable gene detection was performed using the SCTransform function[45]. The bam files generated by SpaceRanger Count v1.3.0 (10x Genomics) were processed via GATK PathSeq v4.1.3.0 Pathogen discovery pipeline (Broad institute, Cambridge, MA, USA)[17] to identify and taxonomically classify microbial reads. Taxa were assigned to reads with a minimum clip length set to 60 bp, and filter-duplicates set to false to avoid loss of duplicated reads. As a result of GATK PathSeq (Broad institute)[17] analysis, a YP tag was added to each microbial read which contains taxa assignments. The Python package Pysam v0.16.0.1 was used to read and process BAM files generated in previous steps. The microbial annotation results were aligned with corrected 10x barcodes (identifying the capture spot of origin) and UMIs (unique transcripts) from SpaceRanger (10x Genomics) output based on the read names. Microbial reads with the highest mapping quality score were used to assign Genera-level taxa identification to corresponding UMIs. The count of microbial UMIs for each spot barcode was summarized into a taxa matrix. The spot-level taxa matrix was attached to the corresponding Visium sample using Seurat's[44] AddMetadata function.

Immunohistochemistry:
FFPE samples were sectioned at 4 µm onto positively charged slides and baked for 1h at 65°C. The slides were deparaffinized using the BOND dewax solution (Leica Microsystem) on a Leica BOND RX stainer platform (Leica Microsystem). Antigen retrieval and antibody stripping steps were

performed at 100°C. Endogenous peroxidase was blocked with 3% H2O2 for 5min followed by protein blocking with TCT buffer (0.05M Tris, 0.15M NaCl, 0.25% Casein, 0.1% Tween 20, 0.05% ProClin300 pH 7.6) for 10 min at room temperature.

**List of primary and secondary antibodies**						
**Protein target**	**Antibody**	**Host/clone**	**Manufacturer/ Cat#**	**Concentration/ Dilution**	**Secondary/ Cat#**	**Opal Dye/ Cat#**
1	EpCAM	Ms/9C4	BioLegend 32402	0.83ug/ml 1:200	Ms PV PV6114	540 FP1494001KT
2	CD66b	Ms/G10F5	BD Biosciences 555723	2ug/ml 1:250	Ms PV PV6114	520 FP1487001KT
3	CD11b	Rb/EP45	BioSB BSB6440	0.5ug/ml 1:250	Rb PV PV6119	620 FP1495001KT
4	CD4	Rb/EP204	CellMarque 104R-26 (AC-0173)	0.15ug/ml 1:60	Rb PV PV6119	570 FP1488001KT
5	CD8	Ms/144B	Dako M7103	0.39ug/ml 1:400	Ms PV PV6114	780 FP1501001KT
1	PD-1	Rb/EPR4877(2)	Abcam ab137132	0.925ug/ml 1:2000	1X Opal Anti-Ms + Rb HRP ARH1001EA	520 FP1487001KT
2	Ki67	Ms/MIB-1	Dako M7240	0.46ug/ml 1:100	1X Opal Anti-Ms + Rb HRP ARH1001EA	570 FP1488001KT
3	CD8	Ms/144B	Dako M7103	0.196ug/ml 1:800	1X Opal Anti-Ms + Rb HRP ARH1001EA	620 FP1495001KT
4	Cytokeratin	Ms AE1/AE3	Dako M3515	0.270ug/ml 1:600	1X Opal Anti-Ms + Rb HRP ARH1001EA	650 FP1496001KT

The first primary antibody against the first protein target (EpCAM) was incubated for 60 minutes, followed by the corresponding secondary antibody incubation for 20min. All secondary antibodies were conjugated with a host-specific, biotin-free, polymeric HRP by using either the PowerVision Poly-HRP anti-Rabbit or PowerVision Poly-HRP anti-Mouse detection antibodies (Leica Microsystem). Tyramide signal amplification (TSA) was performed by incubating with the corresponding Opal fluorophore diluted in the TSA-amplification reagent (PerkinElmer) for 20 minutes. A high-stringency wash was performed following Opal incubation using high-salt TBST solution (0.05M Tris, 0.3M NaCl, and 0.1% Tween-20, pH 7.2-7.6). The primary and secondary antibodies against the first protein target were stripped with the antigen retrieval solution for 20 min before repeating the process against the second protein target (CD66b) starting with a new incubation step with 3% H_2O_2 until all 5 protein targets were completed. Slides were stained with 5 µg/mL DAPI (Sigma), cover slipped with Prolong Gold Antifade reagent and cured for 24h at room temperature in the dark. Whole tissue scans were acquired on a Akoya Vectra Polaris (Akoya Biosciences) at ×20 objective. The images were spectrally unmixed by Phenoptics inForm software (inForm 2.4.8, Akoya, USA). Cell densities in the region of interest were analyzed using the Halo Image Analysis Software

v3.4 (Indica Labs, Albuquerque, NM, USA) applying the Highplex FL module (Indica Labs, Albuquerque, NM, USA.). p-values were calculated using Mann-Whitney test using the GraphPad Prism v7.0 Software (GraphPad Software, La Jolla, CA, USA). Of note, GraphPad Prism v7.0 Software did not report exact p-values when the p-value was <0.0001, as such, any p-value below 0.0001 is reported as <0.0001 in the manuscript.

GeoMx-Digital Spatial Profiling:
FFPE tissue from a total of 18 CRC (10 CRC cases bacteria +ive, 8 CRC cases bacteria -ive) and 8 OSCC patients were profiled using GeoMx-DSP as previously described. RNAscope-CISH images were used to guide the selection of areas of interest (AOIs) that were positive or negative for intratumoral bacteria including *F. nucleatum* in the tumor tissue. In a sequential tissue slide, immunofluorescent visualization marker against Pan-Cytokeratin (PanCK) and CD45 were employed to identify the distribution of the epithelial cancer and immune compartment respectively along the tumor tissue. A multiplexed cocktail of 77 primary antibodies, each with a UV-photocleavable indexing oligonucleotide was incubated on the immunofluorescent slide using the following modules: Immune Cell Profiling Panel, IO Drug Target Panel, Immune Activation Status Panel, Immune Cell Typing Panel, Pan-Tumor Panel, PI3K/AKT Signaling Panel, MAPK Signaling Panel and the Cell Death Panel. Each AOI was exposed with a UV LED light cleaving the associated barcoded oligos from the primary antibodies. The oligos were separately acquired from the immune and epithelial cancer compartment applying the segmented profiling option. The cleaved oligos were collected through microcapillary aspiration for each segment using the GeoMx Digital Spatial Profiler v2.1 Instrument Software and then hybridized to Nanostring designed Tag-set for digital counting using the nCounter analysis system (Nanostring). Using the GeoMx Data Analysis suite software (Nanostring; Version 2.5.0.145) the differential gene expression profile comparing bacteria-positive vs bacteria-negative AOIs per each biological segment was quantified by applying a linear mixed effect model followed by Benjamini-Hochberg multiple correction test providing the fold change and p-values for each gene.

Cell culture invasion assays for scRNA-seq:

<u>HT-29</u>
Cells were seeded at $1.25x10^6$ cells/well into 6-well plates (Nunclon Delta Surface, ThermoScientific) and allowed to adhere for 16 hours. Specific bacterial taxa including *F. nucleatum subsp. animalis COCA36*, *Eschericheria coli DH5alpha*, *Bacteroides fragilis* CTX25T, *Prevotella intermedia 105CP*, *Gemella haemolysans CRC* and *Veillonella parvula CRC* were prepared in in McCoys 5A at an optical density of 1.0 at Abs600 nm and added to HT29 for a final bacterial MOI of 100:1. Co-cultures along with the non-treated HT29 control were incubated for 3 hours at 37°C in 5% CO^2. After incubation, wells were washed five times with PBS with gentle swirling to remove unattached bacteria and HT29 cells. Wells were treated with 0.25% Trypsin-EDTA (Gibco) until cells were lifted, and cells from triplicate wells from the same condition were pooled together in 50 mL conical flasks. An additional 10 mL of McCoys 5A was added to each condition to halt trypsin digestion. Cells centrifuged in a swinging bucket rotor at 250 rcf for 5 minutes at room temperature. The resulting pellets were resuspended in 1 mL of McCoys 5A and gently resuspended prior to the addition of another 2 mL of McCoys 5A and cell filtration through a 70 µm MACS SmartStrainer to remove clumps. Filters cell suspensions were centrifuged at 150 rcf for 3 minutes, and the supernatant removed. Using a wide-bore pipette tip, each pellet was resuspended with 1 mL of PBS + 0.4% BSA. This washing process was repeated twice. The final pellet was resuspended in 500 µl PBS + 0.4% BSA and the cell number and viability measured on a Countess II FL cell counter (Invitrogen). Cells were diluted to 700 cells/µl for each condition.

HCT 116

Cells were seeded at 1.25×10^6 cells/well into a 6-well plate (Nunclon Delta Surface, ThermoScientific) and allowed to adhere for 16 hours. Individual bacterial resuspensions for *F. nucleatum subsp. animalis COCA36*, *Porphyromonas gingivalis W83*, and *Prevotella intermedia 105CP* were prepared in McCoys 5A at an optical density of 1.0 at Abs600 nm. Each bacterial species was added to HCT 116 cells in triplicate wells at a MOI of 100:1 and 500:1. These bacterial–eukaryotic co-cultures were incubated for 3 hours at 37°C in 5% CO^2. The bacterial naïve control HCT 116 cells were also incubated under the same conditions. After incubation, wells were washed four times with PBS with gentle swirling to remove unattached bacteria and HCT 116 cells. Wells were treated with 0.25% Trypsin-EDTA (Gibco) until cells were lifted, and cells from triplicate wells from the same condition were pooled together in 50 mL conical flasks. An additional 10 mL of McCoys 5A was added to each condition to halt trypsin digestion. Cells centrifuged in a swinging bucket rotor at 250 rcf for 5 minutes at room temperature. The resulting pellets were resuspended in 1 mL of McCoys 5A and gently resuspended prior to the addition of another 2 mL of McCoys 5A and cell filtration through a 70 µm MACS SmartStrainer to remove clumps. Filters cell suspensions were centrifuged at 150 rcf for 3 min, and the supernatant removed. Using a wide-bore pipette tip, each pellet was resuspended with 1 mL of PBS + 0.4% BSA. This washing process was repeated twice. The final pellet was resuspended in 500 ul PBS + 0.4% BSA and the cell number and viability measured on a Countess II FL cell counter (Invitrogen). Cells were diluted to 700 cells/µl for each co-culture condition. For the cell preparations for MOI 100 and 500 the following were added together equally prior to scRNA-seq: HCT 116, *F. nucleatum subsp. animalis COCA36*, *Porphyromonas gingivalis W83*, and *Prevotella intermedia 105CP*.

Patient tissue processing to single cells:

Fresh tumor tissue was obtained with consent from patients diagnosed with oral cavity squamous cell carcinoma (OSCC) and undergoing surgical resection at the University of Washington Medical Center. 1-4 mm tissue was cut into small pieces in preparation for single cell generation using a sterile scalpel in a petri dish. Tissue was dissociated following the protocol for the Miltenyi Biotec Human Tumor Dissociation Kit. Briefly, diced tissue was added to RPMI 1640 medium (Gibco) containing the provided enzymatic enzymes in a gentleMACS C tube and loaded onto a gentleMACS Octo Dissociator (OctoMACS) with Heaters and dissociated using the program: 37C_h_TDK_3. The resulting cell suspension was applied to a pre-wetted 70 µm MACS SmartStrainer to remove clumps and the strainer washed with additional RMPI 1640 medium. The filtered cell suspension was pelleted at 300xg for 7 min, followed by treatment with a red blood cell lysis solution (Miltenyi Biotec) for 10 min. Following incubation, cells were washed with DBPS containing 0.04% Ultrapure bovine serum albumin (BSA; Invitrogen), pelleted at 300xg for 10 min, followed by resuspension in 500 µl DBPS containing 0.04% BSA. Cells count and viability were then quantified using a Countess II FL cell counter (Invitrogen).

Single-Cell RNA-seq library preparation and sequencing:

Single-cell RNA-seq libraries were prepared using the Chromium Next GEM Single Cell 5' Kit v2 from 10X Genomics, following the manufacturer's instructions. In brief, single cells were diluted in PBS containing 0.4% BSA to a final concentration of ~700-1,200 cells/µl as determined by a Countess II FL cell counter. A total of 1×10^4 cells were mixed together with reverse transcription (RT) master mix and a 16S reverse primer (1100R: GGGTTGCGCTCGTTG ; final concentration 16 µM). This mixture was loaded together with the Single Cell 5' Gel Beads and partitioning oil into a Chromium Next GEM Chip K and loaded onto a Next GEM Chromium Controller to generate Gel Beads-in-emulsion (GEMS). GEMS were then reverse transcribed in a Mini Amp Plus Thermal Cycler (Applied

Biosciences) programmed at 53°C for 45 min, 85°C for 5 min and held at 4°C. GEMS were then broken and cDNA isolated and purified using Dynabeads MyOne SILANE. Cleaned cDNA was then amplified on the thermocycler programmed at 98°C for 45 sec followed by 11-13 cycles of 98°C for 20 sec, 63°C for 30 sec, 72°C for 60 sec, followed by a final extension at 72°C for 60 min and held at held at 4°C. Amplified cDNA was cleaned using SPRIselect reagent (Beckman Coulter). Resulting cDNA was quantified and quality checked using an Agilent 4200 TapeStation (Agilent Technologies).

10x Genomics 5' gene expression (GEX) library:
Amplified cDNA was enzymatically fragmented, end repaired, and A-tailed followed by a double-sided size selection using SPRIselect beads. Adapter Oligos were then ligated onto the amplicon and PCR-amplification was used to add sample-indexing primers followed by size-selection. GEX libraries were sequenced by an Illumina NextSeq 2000 sequencer with 150 bp paired-end reads using a P3-100 flow cell at 26x10x10x90 or NovaSeq 6000 system. Sequencing data was acquired on the NextSeq 1000/2000 System Suite v1.2.0 (Illumina, Inc.).
.

INVADEseq bacterial 16S rRNA gene library:
Starting with 2-10 µl of amplified cDNA generated with 10x Genomics Chromium Single Cell kit CG000086-RevJ (10x Genomics, Pleasanton, CA), a first round of bacterial 16s enrichment was performed using custom primers 16s_Enrich_Forward (5'AATGATACGGCGACCACCGAGA TCTACACTCTTTCCCTACACGACGCTC3') and 1061R (5'GTGACTGGAGTTCAGACGTG TGCTCTTCCGATCTTCACGRCACGAGCTGACGAC3') at 1 µM (each) and using 10x Genomics Amplification Master Mix (PN 220125) and cDNA Additive (PN 220067). Cycling conditions were as follows: initial denaturation at 98ºC for 45 sec; 35 cycles of denaturation at 98ºC for 20 sec, annealing at 67ºC for 30 sec with a ramp rate of 2ºC/sec, and elongation at 72ºC for 1 min. The final cycle was followed by extension at 72ºC for 1 min. Following amplification, enrichment products were purified with a 0.8x SPRI selection using SPRIselect Reagent (Beckman Coulter, Brea, CA). Purified enrichment products were subjected to size selection with Blue Pippin (Sage Sciences, Beverly, MA, USA) using 1.5% Agarose Gel Cassettes and targeting 955 bp-1215 bp. Using the entire volume of the size selected first enrichment, a second enrichment was performed using the same primers and overall cycling conditions, however, with twenty cycles. Enrichment products were purified as before and then assayed on an Agilent Tapestation 4200 using the Agilent D5000 ScreenTape to measure enrichment product concentrations (Agilent Technologies). Based on D5000 data, 50 ng of the second enrichment product was input into the Indexing Reaction. Indexing was performed per the 10x Genomics Single Cell protocol CG000086-RevJ, using 15 cycles. Importantly, indexed libraries were purified with a modified protocol, using a single-sided 0.8x SPRI selection. Purified and indexed libraries were again subjected to size selection using the same Pippin conditions. Library size distributions were validated using the Agilent High Sensitivity D5000 ScreenTape. Additional library QC, blending of pooled indexed libraries, and cluster optimization was performed using the KAPA Quantification Kit for Illumina (Roche Sequencing and Life Science, Wilmington, MA). Sequencing was performed on a MiSeq (lllumina, Inc., San Diego, CA, USA) employing a paired-end, 300 base read length (PE300), using V3 reagents and multiplexing between 7-9 samples per flowcell. On-instrument secondary analysis was performed with MiSeq Reporter Software v2.5.1 (Illumina, Inc.) using basecalls and quality scores generated by Real-time Analysis (RTA) v1.18.54 (Illumina, Inc.).

Confocal microscopy of intracellular bacteria in cell lines and dissociated patient tissue:
Optical imaging of HCT-116 CRC epithelial cells with bacterial strains was performed with confocal laser scanning microscopy. Cells were washed three times with PBS and fixed in 4% paraformaldehyde

in PBS for 30 min at RT. Following fixation, cells were washed three times in PBS and then permeabilized with 0.2% (*v/v*) Triton X-100 in PBS for 4 mins at RT. Cells were washed three times in PBS and then stained for 20 min at RT with two drops/mL of NucBlue Fixed Cell Strain ReadyProbes (Invitrogen, Carlsbad, CA, USA) and ActinGreen 488 Ready Probes (Invitrogen) to stain DNA and actin, respectively. The membranes of the bacteria in this suspension were stained with 5 µg/mL FM 4-64FX (Molecular Probes). Cells were washed three times with PBS and 10 µl was mounted onto glass slides under a coverslip. Samples were viewed with a Leica SP8 confocal laser-scanning microscope (Leica, Wetzlar, Germany) for image acquisition. Representative confocal micrographs of 512 x 512 pixels (pixel size: 103.3 nm) were acquired and assembled using Fiji (Bio-Formats Plugin installed).

Fresh patient tissue embedding in optical cutting temperature compound (OCT):
Fresh patient tissue was cut as close to 6.5 mm width x 6.5 mm length as possible using sterile scalpels. The bottom of a 15 mm x 15 mm x 5 mm Tissue-Tek cryomold (Sakura Finetek USA, Torrance, CA) was filled with clear OCT compound (Fisher, Hampton, NH), tissue placed in the middle oriented top-side up, and covered with OCT. The cryomold was slowly submerged in a slurry of 2-methylbutane (Honeywell, Charlotte, NC) pre-chilled in a polypropylene beaker submerged in liquid nitrogen. The resulting frozen OCT block was stored at -80°C until sectioning.

Bioinformatic analysis of single cell RNAseq data:

<u>Processing of 10x Genomics 5' scRNAseq GEX data:</u>
Raw fastq files were processed by CellRanger v6.1.1 (10x Genomics) and aligned to human reference genome GRCh38. In the downstream analysis, Unique Molecular Identifiers (UMI) count matrix was processed by Seurat 4.0.4[46,47]. Transcripts were detected in at least three cells, and cells containing at least 200 transcripts were processed for downstream analysis. Cells from different samples were labeled by sample identifiers and merged using the Merge function. Next, the top 5,000 variable genes across merged samples were set as the variable features. Then the linear transformation was performed on the merged data based on all genes. After the linear transformation, the linear dimensional reduction (PCA) was performed based on previously defined variable features. Harmony v1.0 is an R package integrating single-cell datasets[48]. After PCA, Harmony[48] was used to integrating merged samples based on sample identifiers. Once Harmony integrated the dataset, UMAP non-linear dimensional reduction was performed using the first 20 dimensions by applying the Harmony dimensional reduction as the input and random seed was set to '111'. Then the nearest neighborhoods for the dataset were computed by FindNeighbors function using the first 20 dimensions, using Harmony dimensional reduction option and then cells were clustered using FindClusters function with the resolution of 0.5.

SingleR v1.4.1 is an R package annotates single-cell data with cell types according to the gene expression similarity between transcriptional cell groups and reference datasets[49]. In this study, SingleR was used for annotating predicted cell types to each cell cluster of the integrated and clustered single cell dataset using the main and the fine label of Human Primary Cell Atlas Data as the reference[50]. To determine the marker genes for each cell population for the myeloid and epithelial-mesenchymal compartment (**Extended Data Fig. 6a**), we have selected the top 5 genes that were the most differentially regulated for each cell cluster in relation with the rest of the cell clusters. For the T cell annotations the following list of marker genes[51] was used as an input to determine each T cell population: *CD3D*, *CD3E*, *CD3G*, *CD4*, *CD40LG*, *CD8A*, *CD8B*, *SELL*, *CCR7*, *NR4A1*, *ROCKC*, *TBX21*, *CCR6*, *CXCR3*, *IL17A*, *IFNG*, *CXCR5*, *PDCD*1, *FOXP3*, *CTLA4*, *SLC4A10*, *TRAV1-2*, *CX3CR1*, *GZMB*, *GZMH*, *GNLY*, *PRF1*, *CRTAM*, *GZMK*, *CCR9*, *ITGAE*, *ITGA1*, *SPRY1*, *SPRY2*,

TRDC, *ITGAD*, *KIR2DL4*, *IKZF2*, *MKI67*, *TYROBP*, *NCAM1*, *FCGR3A*, *CD160*, *PCDH9*, *KIT* and *LST1*. After running the analysis with these marker genes in the T cell compartment (CD3E positive cells), we have identified the following T cell populations: Tissue resident memory CD8+ T cells with the expression of *CD8A* and *CD8B*, conventional cytolytic genes such as *GZMB*, *GZMH*, *GNLY*, *PRF1*, *CRTAM* and *GZMK* with expression of tissue-homing molecules such as *ITGAE* and *ITGA1*. A regulatory CD4+ T cell population that express *CTLA4* and *FOXP3*. Effector memory CD8+ T cells expressing conventional CD8 markers along with cytolytic genes, but they do not express the tissue-homing molecules. Central memory CD4+ T cells that expressed high levels of *CD62L* (*SELL*) and *CCR7* compared to the Effector memory CD4+ T cell population. Seurat v4.0.4 and ggplot2[52] were used to generate dimensional reduction plots using DimPlot function. For data visualization we used FeaturePlot function, and DotPlot function to generate the dot plots (**Extended Data Fig. 6a**).

Differential expression analysis between cellular groups was performed using the FindMarker function in the Seurat package with the Wilcoxon Rank Sum test. The log-fold change is calculated based on the difference of average gene expression between the two groups. P-value is adjusted based on Bonferroni correction with all genes in the dataset. In this study, the results contained only genes that were detected in at least 10% of the cells in either of the cell groups and with log-fold change higher, lower, or equal to 0. Then the differential expression analysis output was used as the input for the volcano plots.

The list of genes from differential expression analysis was then ranked based on the log fold change and then processed with Gene Set Enrichment Analysis (GSEA) using ClusterProfiler v3.18.1 [53] to determine which pathways in the Hallmark gene sets were statistically significantly enriched[54]. All gene sets from the Hallmark Collection were included in the GSEA analysis, and the boundary for calculating the p-value (`eps` argument) was set to zero for better estimation. Enriched Hallmark pathways with p-values lower than 0.05 were reported as significantly enriched pathways.

CopyKAT v1.0.5 (Copy number Karyotyping of Tumors)[55] is an R package that predicts tumor cells from single-cell datasets using integrative Bayesian approaches. The prediction by CopyKAT is based on the idea that cells with a high volume of genome-wide copy number variation are considered tumor cells. In contrast, non-tumor cells usually contain approximately 2N diploid copy numbers. CopyKAT was used to generate a metadata matrix for integrated single-cell samples, which annotates diploid cells and aneuploid cells. The CopyKAT prediction was then mapped to single-cell data using the AddMetadata function in Seurat[44].

<u>Identification of microbial reads within single cells GEX libraries:</u>
Following standard 10x Genomics 5' cDNA library preparation and sequencing on a NovaSeq, the bam files generated by CellRanger v6.1.1 (10x Genomics) were processed via GATK PathSeq (Broad institute) to identify microbial reads as described previously[17]. The taxa were assigned for each read with minimum clip length set to 60 bp and filter-duplicates set to false to avoid loss of duplicated reads. YP tag is added to each microbial read which contains taxa assignments. As previously described Pysam was used to read and process BAM files. Then pathogen annotation was aligned with the corrected barcode and UMI from the CellRanger (10x Genomics) output bam file based on the same read names. Genera-level taxa was assigned to corresponding UMI based on the microbial read with the highest mapping quality score. The count of microbial UMI for each cell barcode was summarized into a taxa matrix. After sample identifiers were added to cell barcodes, matrixes for all the samples were merged using Pandas 0.25.3[56].

INVADEseq bacterial 16S rRNA gene libraries:
Following sequencing on a MiSeq, raw fastq files were processed by CellRanger v6.1.1 (10x Genomics) to obtain corrected barcode and corrected UMI for each read based on 'UB' and 'CB' tag from CellRanger bam files. Then the bam files were converted to fastq format using Bedtools v2.29.2[57], the first 15 bases were cropped from read one fastq files, then TruSeq3 PE adapter sequences and low-quality bases were trimmed using Trimmomatic v0.39 (Institut Pasteur, Paris, France)[58]. Trimmed read one fastq files were then converted to ubam files using Picard 2.21.6 (Broad Institute, 2019). The taxa were assigned for each read using GATK PathSeq (Broad institute) [17], with minimum clip length set to 60 bp and filter-duplicates set to false to avoid loss of duplicated reads. Then, Pysam was used to read and process BAM files generated in previous steps. Microbial taxa resolution was set to genera and annotated reads were aligned with corrected barcodes and UMIs from CellRanger (10x Genomics) output based on read names. The genera-level taxa were assigned to corresponding UMI based on the read with the highest mapping quality score. The count of microbial UMI for each cell barcode was summarized into a taxa matrix. After sample identifiers were added to cell barcodes, matrixes for all the samples were merged using the Pandas package. The final INVADEseq taxa matrix was generated using the Pandas package to combine the GEX taxa matrix and INVADEseq taxa matrix. The cellular-level taxa matrix was attached to the integrated single-cell sample using the AddMetadata function in Seurat[44]. Differential expression analysis between cellular groups was performed using the FindMarker function in the Seurat[44] package based on the Wilcoxon Rank Sum test. The log-fold change is calculated based on the difference of average gene expression between two groups. P-value was adjusted based on Bonferroni correction with all genes in the dataset. The threshold for adjusted p-value was set to 0.05 for significantly regulated genes between two groups. Significantly regulated genes were then ranked based on log fold change and then processed with Gene Set Enrichment Analysis (GSEA)[59] using ClusterProfiler[53] package to determine which pathway in the "Hallmark" gene set were statistically significantly enriched. Enriched pathways with a p-value lower than 0.05 were reported as significantly enriched pathways.

Single cell RNAseq data analysis approach:
In the HCT-116 experiment described in the Extended Data Figure 4d, by using the UMI metric we can select cancer cells that contain a higher number of bacterial transcripts by applying different bacteria UMI cutoffs. In the absence of any UMI cutoff we found a weak gene signature when comparing total cancer cells associated with either total *Fusobacterium nucleatum* (Total *Fuso*+) or *Porphyromonas gingivalis* (Total *Porph*+) against total uninfected cells (Total Bac-) "All clusters cell analysis". However, after applying a ≥3 bacteria UMI cutoff, we could select the cancer cell clusters that are associated with bacteria exposure, and they are transcriptionally different from the other cell populations in the cell line (**Extended Data Fig. 5a-d**). Therefore, cancer cells that were weakly interacting with *F. nucleatum* or *P. gingivalis* were removed from the analysis improving the gene expression profile exhibiting a higher number of differentially expressed molecules among conditions (Bacteria single cell analysis; **Extended Data Fig. 5a-d**). When the analysis was performed among specific cell cluster "Specific cell cluster analysis", for instance, *F. nucleatum*-associated cells from cluster 5 or *P. gingivalis*-associated cells from cluster 6 against bacteria negative cells from cluster 1 (control cell cluster) we found a clearer expression signal since the comparisons were made between cell population from different transcriptional groups or cell clusters.

A similar approach was implemented using the integrated data from OSCC patients where a ≥3 bacteria UMI cutoff could eliminate weakly interacting *Fusobacterium* or *Treponema* selecting a transcriptional group of cells that is associated with these microorganisms thus improving the gene expression signal (**Fig. 3d-g**). At individual patient level, samples with higher bacteria load are

associated with bacterial species that belong to the *Fusobacterium* and *Treponema* genera and it correlates with a strong inflammatory response (**Extended Data Fig. 8**). Therefore, samples with higher bacteria load are correlated with an increased infiltration of immune cells obscuring the gene expression signal from other cell populations in which the epithelial cluster is almost absent in some cases. As the bacteria load is lower, the expression inflammatory genes are less pronounced allowing the expression of genes that are more commonly found in epithelial cells such as *SERPIN* molecules and other oncogenes such as *SPARC*, *PARD3B*, *TAGLN* and *TPM*1. Since each patient sample had a different cellular composition, the macrophage and epithelial cell cluster are not completely defined and therefore we could only analyze the gene expression profile in the entire sample "All cluster analysis" comparing bacteria-associated cells from the most dominant bacterial species against total uninfected cells for each sample (**Extended Data Fig. 8**). In this type of analysis, the gene expression profile and corresponding GSEA are derived from all cell populations, not specific annotated cell types, from the same tumor tissue. To overcome this issue, data integration allows us to increase the statistical power combining the number of cells for each cell population from all samples thus generating more defined cell clusters (**Fig. 3c**). In this manner, we could separate the gene expression signal from the immune and epithelial compartment by analyzing the differential gene expression and GSEA in each individual cell cluster including the macrophage and epithelial cell clusters from the integrated data (**Fig. 3d-g**; "Specific cell type cluster analysis").

Viral transduction:
Lentiviral particles containing the Lifeact-GFP[60,61] construct were manufactured by ibidi GmbH (Martinsried, Germany). A total of $2x10^4$ HCT-116 cells were seed in 4 wells from 24-well plates the day before viral transduction in DMEM culture media. When the cell culture reached 30-50% confluence the culture medium was replaced with 500µl of culture media containing 10 µl of lentiviral particles (Viral titer = 1x 10^7 transducing units (TU)/ml), which corresponded to a multiplicity of infection (MOI) of 5. A total of 4 µl of polybrene (1mg/ml aliquoted) was added in each well for a final concentration of 8 µg/ml. For an enhanced transduction efficiency, the 24 well-plate was centrifuged at 800g for 90min and then it was incubated overnight at 37°C and 5% CO^2. Following incubation, the viral culture media was replaced with standard DMEN media and the LifeactGFP fluorescent signal was detected after 3 days of transduction. LifeactGFP positive cells were selected by the addition of 5 µg/ml puromycin-containing medium every 3 days for two weeks. Resistant colonies were eliminated by using FACS-sorting reaching purities above 90%.

Bacterial preparation for spheroid and cluster cell formation:
Fusobacterium nucleatum subsp. animalis COCA36 colonies were picked using a sterile cotton swab and suspended in complete DMEN culture media without Pen/Strep to an optical density of 1.0 at an absorbance (Abs) of 600 nm (approximately $5x10^8$ colony forming units (CFU) per mL). Bacterial cells were pelleted by centrifugation at 8000 rcf for 3 min and incubated with 5 µg/mL FM 4-64 FX membrane dye (Thermo Fisher Scientific, Waltham, MA, USA) resuspended in 1 mL of PBS for 20 min in the dark at room temperature. Following three washes with PBS, the bacterial cell suspension was diluted and added to wells containing human cells to generate cocultures at a multiplicity of infection (MOI) of 100:1, 10:1, and 1:1.

3D Invasion assay:
To form spheroids a total of $2x10^5$ human HCT-116 or mouse CT26WT CRC cells expressing the Lifeact-GFP construct were seeded in ultra-low attachment 96-well plates (Corning, New York, NY, USA) treated with or without *F. nucleatum* stained with 5 µg/mL FM 4-64 FX stain (Thermo Fisher Scientific) at MOI of 100 for 12h incubation period. The CRC spheroids per each experimental

condition were embedded in a liquid-phase rat-tail collagen solution (50 µl at ~3 mg/ml; Corning) containing 1N NaOH (1.14µl) and 10× PBS (10 µl) for a total volume of 100µl on ice. A volume of 70µl of the solution was rapidly transferred to a Lab-Tek II chambered cover glass with a #1.5 borosilicate glass bottom (Thermo Fisher Scientific) and incubated at 37°C and 5% CO^2 for 10 min to allow the gel to polymerize into a 3D collagen matrix. After gel solidification a volume of 500 µl were added to the top of the collagen matrices. Then, the Lab-Tek chambers were place on a confocal microscope for live-cell imaging.

Neutrophil swarming assay:
Neutrophils derived from the HL-60 cell line were labelled with 5 µM CMTMR dye (Thermo Fisher Scientific) and embedded in 50 µl of a collagen solution (see 3D Invasion assay) on ice and resuspended with another 50µl of collagen solution containing CRC spheroids expressing the GFPLifeact construct previously treated with or without *F. nucleatum* at MOI of 100 in ultra-low attachment 96-well plates for 12h incubation period. From the collagen mix a total of 70µl were transferred to a Lab-Tek II chambered cover glass with a #1.5 borosilicate glass bottom (Thermo Fisher Scientific) and incubated at 37°C and 5% CO^2 for 10 min to allow the gel to polymerize into a 3D collagen matrix. After gel solidification a volume of 500 µl were added to the top of the collagen matrices. Then, the Lab-Tek chambers were place on a confocal microscope for live-cell imaging.

Live-cell confocal imaging:
Imaging data was recorded in a four-dimensional space through a 20x water immersion objective, numerical aperture (NA) of 1.37 (Leica Microsystems, Wetzlar, Germany) using a Leica TCS SP8 confocal microscope equipped with a resonant scanner and an incubator that maintains 37^0C and 5% CO2 during the imaging period. LifeactGFP expression was exited at 488nm, and the FM 4-64 FX membrane dye was excited at 565nm using a tunable white light laser (Leica Microsystem). Imaging data was acquired on the Leica Application Suite X (LAS X; Leica Microsystem). Images were obtained with a total z-thickness of 70 µm taking a step size every 2 µm. For neutrophils movement, each z stack was collected every 2 min and for the invasion assays using the CRC cell lines HCT-116 or CT26WT every 10min for a total imaging period of ~20h.

Imaging analysis:
HL-60 neutrophil movements were quantified inside CRC spheroids derived from the HCT-116 cells line expressing the LifeactGFP construct. Using Imaris Cell Imaging Software v9.2.0 software (Bitplane AG, Zurich, Switzerland) neutrophils were segmented using the CMTMR signal thus creating surfaces with a filter below 100µm^3 to remove cell debris in the analysis. Differentiated CMTMR-neutrophils were tracked inside the boundaries of the LifeactGFP signal from the CRC spheroids using an autoregressive motion model, applying a threshold of 600sec to exclude tracks with insufficient duration in the imaging space. Morphological and tracking data were exported providing different migration parameters to quantify population wide motility behaviors in HL-60 cells inside CRC spheroids. Cell volume dynamics was quantified as a measurement of neutrophil cluster formation inside CRC spheroids infected with or without *F. nucleatum* embedded in collagen matrices. For each experimental condition the cell volumes were calculated relative to the volumes at the beginning of the experiment (T=0). The area under curve was calculated for each experimental condition for positive cell volume changes above baseline (y=0). For the invasion assays, the expansion rate of uninfected CRC spheroids was quantified by measuring the slopes of the fit straight lines from the non-normalized plots of the volume changes over time from uninfected spheroid masses embedded in collagen matrices using the LifeactGFP fluorescent signal as a read out. For *F. nucleatum* infected spheroids, the invading CRC cells were tracked using the FM 4-64 FX membrane dye signal

of infecting migrating cells that were masked outside the LifeactGFP signal from the spheroid masses. p-values were calculated by applying Mann-Whitney test using GraphPad Prism v7.0 Software (GraphPad Software). Note: Prism software does not report exact p values below 0.0001 and these values are reported as $p < 0.0001$.

Cell cluster formation assay:
A total of $2x10^5$ HCT-116 cells expressing the LifeactGFP construct or HL-60 cells expressing the soluble GFP were co-cultured with a human CRC *F. nucleatum* isolated labeled with 5 µg/mL FM 4-64 FX membrane dye at multiplicity of infection (MOI) at 0, 1, 10 and 100 for 2h in ultra-low attachment 6 well plates (Corning). A total of 500µl of cell suspension for each experimental condition was transferred into the Lab-Tek II imaging chamber for confocal imaging (Leica TCS SP8) using the 20x water immersion objective. For each independent experiment, three fields of view were collected for each experimental condition using either human cell line with a z-thickness between 70 to130µm and 2µm step size. Using Imaris software the cell objects were masked using the GFP fluorescence signal providing morphological characteristics including the number and size (volume μm^3) of each cell object from each condition in ether cell line.

Flow cytometry:
For intracellular staining against phosphoproteins, HCT-116 and HL-60 cells were incubated with a viability marker implementing the LIVE/DEAD fixable Aqua Dead cell staining (ThermoFisher Scientific) for 30min on ice. Following incubation, the cells were fixed for 30min at room temperature in the dark and then permeabilized using the BD Cytofix/Cytoperm plus kit (BD Biosciences). The permeabilization solution contained a cocktail of antibodies against phosphoproteins including 1.25µg/ml Phospho-p44/42 MAPK (Erk1/2) (Thr202/Tyr204) (E10) Mouse mAb (Alexa Fluor 488 conjugate) and 1.25µg/ml Phospho-p38 MAPK (Thr180/Tyr182) (3D7) Rabbit mAb (Phycoerythrin conjugate). Following 45min of incubation with the anti-phospho antibodies on ice, the permeabilization solution was washed twice with FACS wash buffer (2% HI-FCS, 2 mM EDTA and 0.02% sodium aside in 1x PBS). Final cell suspensions were prepared in 200µl cold FACS wash buffer and $1x10^4$ viable cell events (LIVE/DEAD aqua negative cells) were acquired on the BD Fortessa x20 flow cytometer for each experimental condition (BD Biosciences, Franklin Lakes, NJ, USA). A tube only containing *F. nucleatum* was also acquired to gate-out undesired bacteria cell events from the analysis. From the live cell population (Live/Dead aqua unstained cells) a total number of $1x10^4$ live cells were collected using the FACS Diva Software v5.0.2 (BD Biosciences). The percentages of positive fluorescent cells were calculated for each phosphoprotein and for each experimental condition relative to an unstained control condition. Flow cytometry data were analyzed with FlowJo V9 software (Treestar Inc., Ashland, OR, USA).

RNA extraction and hybridization to nCounter code-set:
CRC spheroids derived from the human HCT-116 or mouse CT26WT cell line were treated with or without *F. nucleatum* at MOI=100 for 12h at 37°C and 5% CO^2. Spheroids were lysed for RNA extraction by adding 350µl of lysis buffer into the spheroid pellets. After addition of 750µl of 70% ethanol the solution was transferred to a RNeasy Mini spin column (RNeasy Mini RNA Isolation Kit; Qiagen, Hilden, Germany). Contaminant DNA genomic was digested by adding 80 µl of DNase I solution into the dried columns for 15 min at room temperature. Following several cycles of washes the RNA was eluded in 30 µl of H_2O_2 and collected in 1.5 ml tubes. RNA integrity and concentration were measured by using Agilent 4200 Tapestation (Agilent Technologies, Santa Clara, CA, USA) with RNA integrity numbers (RINs) above 9.0 in all samples. For human and mouse samples the RNA was hybridized to the human and mouse nCounter Tumor Signaling 360 Panel (Nanostring, Seattle, WA,

USA) respectively. For each sample, a volume of 5 µl of 20 ng/ml total RNA was mixed with 8 µl of the hybridization master mix containing the reporter codeset and 2 µl of capture probeset for each reaction. The hybridization reactions were incubated for 16 hours and acquired in the nCounter MAX/FLEX platform (Nanostring) following manufacture instructions. Gene expression data was analyzed by using nSolver software (Nanostring) applying background threshold option to discard the counts below the negative control probe counts for each run. Data was normalized against the positive control probes counts and the housekeeping genes from the codeset content. Differential gene expression and pathway analysis was performed using the nSolver™ Analysis Software v.4 (NanoString) with the Advanced analysis plugin (Nanostring; version 2.0.134). Statistically significant, differentially expressed genes were defined as those with expression levels corresponding to a log2 ratio >0.56 or< −0.56 and p-value<0.05.

Reproducibility statement:
A total of 11 patient fresh-frozen colorectal cancer (CRC) tumors were selected for microbiome bulk analysis using 16S ribosomal RNA gene sequencing (**Extended Data Figure 1a, Supplementary Table 1**). RNAscope was implemented to assess the presence of intratumoral bacteria for a total of 23 CRC and 17 OSCC individual patient tumors included in this manuscript (**Figure 1c, Figure 2a, Figure 3a, Extended Data Figure 1e, Extended Data Figure 2a, Extended Data Figure 3a, Extended Data Figure 3c**). Microbiome composition and bacteria distribution using 10x Visium spatial transcriptions were measured in a CRC and oral squamous cell carcinoma (OSCC) case embedded in OCT medium, these cases were selected as they were positive for bacteria via RNAscope FISH (**Figure 1**). An additional 19 CRC and 8 OSCC FFPE embedded individual patient specimens were included for GeoMx-DSP analysis, all specimens had IHC for PanCK, CD45, SMA and DNA, along with RNAscope CISH for *F. nucleatum* and *Eubacteria* performed on a sequential section (**Figure 2a, Extended Data Figure 2b, Extended Data Figure 3e, Supplementary Table 1**). IHC for specific targets noted in **Extended Data Figure 3,** and RNAscope CISH for *F. nucleatum* and *Eubacteria* from a sequential section, were performed on four OSCC and four CRC specimens from 8 patients included in the GeoMx analysis (**Extended Data Figure 3a and Extended Data Figure 3c**). A total of 7 fresh OSCC samples were processed for *INVADEseq* single-cell RNA sequencing (**Figure 3, Supplementary Table 1**). Confocal microscopy to visually assess the presence of intracellular bacteria in cell lines co-cultured with bacteria was performed independently at least three times with comparable results (**Extended Data Figure 4e**). Confocal microscopy to visually assess the presence of intracellular or adherent bacteria in single cells from patient tumors were performed on dissociated single cells from two individual patients and cell associated bacteria were observed in both cases (**Figure 3a**). For *in-vitro* functional assays the experiments were conducted independently at least three times with comparable results each time (**Figure 4a, Figure 4f, Extended Data Figure 9a, Extended Data Figure 9d, Extended Data Figure 9l**).

Code availability:
Custom code for data processing and analysis are available at https://github.com/FredHutch/Galeano-Nino-Bullman-Intratumoral-Microbiota_2022.

Data availability:
Raw sequencing data from bulk 16S ribosomal RNA gene sequencing, 10x Visium spatial transcriptomics and *INVADEseq* bacterial 16S rRNA and 10x genomics 5’ human (GEX) gene libraries are available in the NCBI Sequence Read Archive (SRA) repository under the Bioproject accession number PRJNA811533. PathSeq, Cell Ranger and Space Ranger analyses used GRCh38 as the human genome reference.

References

41 Chong, J., Liu, P., Zhou, G. & Xia, J. Using MicrobiomeAnalyst for comprehensive statistical, functional, and meta-analysis of microbiome data. Nat Protoc 15, 799-821, doi:10.1038/s41596-019-0264-1 (2020).

42 Martin, F. E., Nadkarni, M. A., Jacques, N. A. & Hunter, N. Quantitative microbiological study of human carious dentine by culture and real-time PCR: association of anaerobes with histopathological changes in chronic pulpitis. J Clin Microbiol 40, 1698-1704, doi:10.1128/JCM.40.5.1698-1704.2002 (2002).

43 Castellarin, M. et al. Fusobacterium nucleatum infection is prevalent in human colorectal carcinoma. Genome Res 22, 299-306, doi:10.1101/gr.126516.111 (2012).

44 Hao, Y. et al. Integrated analysis of multimodal single-cell data. Cell 184, 3573-3587 e3529, doi:10.1016/j.cell.2021.04.048 (2021).

45 Hafemeister, C. & Satija, R. Normalization and variance stabilization of single-cell RNA-seq data using regularized negative binomial regression. Genome Biol 20, 296, doi:10.1186/s13059-019-1874-1 (2019).

46 Zheng, G. X. et al. Massively parallel digital transcriptional profiling of single cells. Nat Commun 8, 14049, doi:10.1038/ncomms14049 (2017).

47 Stuart, T. et al. Comprehensive Integration of Single-Cell Data. Cell 177, 1888-1902 e1821, doi:10.1016/j.cell.2019.05.031 (2019).

48 Korsunsky, I. et al. Fast, sensitive and accurate integration of single-cell data with Harmony. Nat Methods 16, 1289-1296, doi:10.1038/s41592-019-0619-0 (2019).

49 Aran, D. et al. Reference-based analysis of lung single-cell sequencing reveals a transitional profibrotic macrophage. Nat Immunol 20, 163-172, doi:10.1038/s41590-018-0276-y (2019).

50 Mabbott, N. A., Baillie, J. K., Brown, H., Freeman, T. C. & Hume, D. A. An expression atlas of human primary cells: inference of gene function from coexpression networks. BMC Genomics 14, 632, doi:10.1186/1471-2164-14-632 (2013).

51 Dominguez Conde, C. et al. Cross-tissue immune cell analysis reveals tissue-specific features in humans. Science 376, eabl5197, doi:10.1126/science.abl5197 (2022).

52 Wickham, H. in ggplot2 : Elegant Graphics for Data Analysis (Springer International Publishing : Imprint: Springer, Cham, 2016).

53 Yu, G., Wang, L. G., Han, Y. & He, Q. Y. clusterProfiler: an R package for comparing biological themes among gene clusters. OMICS 16, 284-287, doi:10.1089/omi.2011.0118 (2012).

54 Liberzon, A. et al. The Molecular Signatures Database (MSigDB) hallmark gene set collection. Cell Syst 1, 417-425, doi:10.1016/j.cels.2015.12.004 (2015).

55 Gao, R. et al. Delineating copy number and clonal substructure in human tumors from single-cell transcriptomes. Nat Biotechnol 39, 599-608, doi:10.1038/s41587-020-00795-2 (2021).

56 McKinney, W. in Proceedings of the 9th Python in Science Conference. 51-56 (Austin, TX).

57 Quinlan, A. R. & Hall, I. M. BEDTools: a flexible suite of utilities for comparing genomic features. Bioinformatics 26, 841-842, doi:10.1093/bioinformatics/btq033 (2010).

58 Bolger, A. M., Lohse, M. & Usadel, B. Trimmomatic: a flexible trimmer for Illumina sequence data. Bioinformatics 30, 2114-2120, doi:10.1093/bioinformatics/btu170 (2014).

59 Subramanian, A. et al. Gene set enrichment analysis: a knowledge-based approach for interpreting genome-wide expression profiles. Proc Natl Acad Sci U S A 102, 15545-15550, doi:10.1073/pnas.0506580102 (2005).

60 Galeano Nino, J. L., Tay, S. S., Tearle, J. L. E., et al. The Lifeact-EGFP mouse is a translationally controlled fluorescent reporter of T cell activation. J Cell Sci 133, doi:10.1242/jcs.238014 (2020).

61 Riedl, J. et al. Lifeact: a versatile marker to visualize F-actin. Nat Methods 5, 605-607, doi:10.1038/nmeth.1220 (2008).
