# Supplementary material for: Effect of the intratumoral microbiota on spatial and cellular heterogeneity in cancer
Source: Nature. 2022 Nov 16;611(7937):810–7. doi: 10.1038/s41586-022-05435-0 (PMC9684076; doi:10.1038/s41586-022-05435-0)
Supplement: Supplementary file 2 — Reporting Summary [file 41586_2022_5435_MOESM2_ESM.pdf]

nature portfolio

Corresponding author(s): Susan Bullman
Christopher D. Johnston

Last updated by author(s): Sep 2, 2022

Reporting Summary

Nature Portfolio wishes to improve the reproducibility of the work that we publish. This form provides structure for consistency and transparency in reporting. For further information on Nature Portfolio policies, see our Editorial Policies and the Editorial Policy Checklist.

Statistics

For all statistical analyses, confirm that the following items are present in the figure legend, table legend, main text, or Methods section.

n/a	Confirmed	
☐	☒	The exact sample size (*n*) for each experimental group/condition, given as a discrete number and unit of measurement
☐	☒	A statement on whether measurements were taken from distinct samples or whether the same sample was measured repeatedly
☐	☒	The statistical test(s) used AND whether they are one- or two-sided<br>*Only common tests should be described solely by name; describe more complex techniques in the Methods section.*
☐	☒	A description of all covariates tested
☐	☒	A description of any assumptions or corrections, such as tests of normality and adjustment for multiple comparisons
☐	☒	A full description of the statistical parameters including central tendency (e.g. means) or other basic estimates (e.g. regression coefficient) AND variation (e.g. standard deviation) or associated estimates of uncertainty (e.g. confidence intervals)
☐	☒	For null hypothesis testing, the test statistic (e.g. *F*, *t*, *r*) with confidence intervals, effect sizes, degrees of freedom and *P* value noted<br>*Give P values as exact values whenever suitable.*
☐	☒	For Bayesian analysis, information on the choice of priors and Markov chain Monte Carlo settings
☐	☒	For hierarchical and complex designs, identification of the appropriate level for tests and full reporting of outcomes
☒	☐	Estimates of effect sizes (e.g. Cohen's *d*, Pearson's *r*), indicating how they were calculated

Our web collection on statistics for biologists contains articles on many of the points above.

Software and code

Policy information about availability of computer code

Data collection	BD FACSDiva™ Software<br>GeoMx Digital Spatial Profiler v2.1 Instrument Software<br>Leica Application Suite X (LAS X)<br>NextSeq 1000/2000 Control Software Suite v1.2.0

Data analysis

FlowJo v9
Imaris Cell Imaging Software (Bitplane) v9.2.0
nSolver™ Analysis Software v.4 (NanoString)
GraphPad Prism 7.0 Software
Halo Image Analysis Software v3.4 (Indica Labs)
R packages: ClusterProfiler v3.18.1, CopyKAT v1.0.5, Harmony v1.0, Seurat v4.0.4, SingleR v1.4.1
Python package: Pysam 0.16.0.1
CellRanger v6.1.1 (10x Genomics)
GATK PathSeq v4.1.3.0 Pathogen discovery pipeline (Broad institute)
SpaceRanger v1.3.0 (10x Genomics)
Trimmomatic v0.39
MicrobiomeAnalyst

For manuscripts utilizing custom algorithms or software that are central to the research but not yet described in published literature, software must be made available to editors and reviewers. We strongly encourage code deposition in a community repository (e.g. GitHub). See the Nature Portfolio guidelines for submitting code & software for further information.

Data

Policy information about availability of data

All manuscripts must include a data availability statement. This statement should provide the following information, where applicable:

- Accession codes, unique identifiers, or web links for publicly available datasets
- A description of any restrictions on data availability
- For clinical datasets or third party data, please ensure that the statement adheres to our policy

Codes for data processing and analysis is available at https://github.com/FredHutch/Galeano-Nino-Bullman-Intratumoral-Microbiota-2022. Raw sequence data from microbiome analysis using bacterial 16S ribosomal RNA gene sequencing, 10x Visium spatial transcriptomics and single-cell RNA sequences were deposited in the NCBI Sequence Read Archive (SRA) under the Bioproject accession number PRJNA811533. PathSeq, Cell Ranger and Space Ranger analyses used GRCh38 as the human genome reference.

Field-specific reporting

Please select the one below that is the best fit for your research. If you are not sure, read the appropriate sections before making your selection.

☒ Life sciences ☐ Behavioural & social sciences ☐ Ecological, evolutionary & environmental sciences

For a reference copy of the document with all sections, see nature.com/documents/nr-reporting-summary-flat.pdf

Life sciences study design

All studies must disclose on these points even when the disclosure is negative.

Sample size

Patient specimen sample size was determined according to biological material available. For the GeoMx analysis, 12 areas of interest were selected per patient specimen and patient cohorts were analyzed independently as stated in the figure legends and methods. For the INVADEseq approach validation with the HCT116 cell line (MOI=0,100 and 500) we captured a total of 12,412 single cells and all cells that passed quality filtering (as detailed in the data exclusions) were included in the analysis . For the INVADEseq approach with the patient OSCC specimens, 42,810 single cells were captured from seven patients, all cells that passed quality filtering (as detailed in the data exclusions) were included in the analysis. For the 10x Visium OSCC and CRC cases, data was obtained from 3404 and 3106 capture spots within the tissue area. Statistical tests were performed for all the analyses as indicated in the figure legends and methods.

Data exclusions

For single-cell RNA sequencing, low quality events were removed by filtering transcripts that were expressed in less than three cells (min.cells feature from Seurat R package) and cells that contain less than 200 unique transcripts (min.feature option from Seurat R package). For the INVADEseq approach validation with the HCT116 cell line (MOI=0,100 and 500), of the total 12,412 single cells captured, 39 single cells were excluded as they did not pass the quality filter as detailed in the methods. For the INVADEseq approach with the seven patient OSCC specimens, of 42,810 total single cells captured, 1087 single cells did not pass the quality filters (as detailed in the methods) and were excluded from analyses. For 10x visium spatial transcriptomics, capture spots that contain less than 3 unique transcripts were removed from the analysis by min.feature option from Seurat R package.

Replication

A total of 11 fresh-frozen colorectal patient cancers (CRC) samples were selected for microbiome bulk analysis using 16S ribosomal RNA gene sequencing. Microbiome composition and bacteria distribution using 10x Visium spatial transcriptions were measured in a CRC and oral squamous cell carcinoma (OSCC) case embedded in OCT medium. An additional 18 CRC and 8 OSCC FFPE embedded samples were collected for GeoMx-DSP analysis. A total of 7 fresh OSCC samples were processed for single-cell RNA sequencing. For in-vitro functional assays the experiments were conducted at least three times for data reproducibility. All replicates successfully show consistent results.

Randomization

Sample randomization into experimental groups are not relevant, as the design of the study aims to quantify discriminative features between already established groups.

Blinding

Blinding is not relevant for this study since the aim of this work is to quantify discriminative features between already establish experimental

Blinding	biological groups.

Reporting for specific materials, systems and methods

We require information from authors about some types of materials, experimental systems and methods used in many studies. Here, indicate whether each material, system or method listed is relevant to your study. If you are not sure if a list item applies to your research, read the appropriate section before selecting a response.

Materials & experimental systems

n/a	Involved in the study
☐	☒ Antibodies
☐	☒ Eukaryotic cell lines
☒	☐ Palaeontology and archaeology
☒	☐ Animals and other organisms
☐	☒ Human research participants
☒	☐ Clinical data
☒	☐ Dual use research of concern

Methods

n/a	Involved in the study
☒	☐ ChIP-seq
☐	☒ Flow cytometry
☒	☐ MRI-based neuroimaging

Antibodies

Antibodies used

anti-EpCAM, Mouse mAb, BioLegend, Cat: 32402, Clone: 9C4
anti-CD66b, Mouse mAb, BD Biosciences, Cat: 555723, Clone: G10F5
anti-CD11b, Rabbit mAb, BioSB, Cat: BSB6440, Clone: EP45
anti-CD4, Rabbit mAb, CellMarque, Cat: 104R-26 (AC-0173), Clone: EP204
anti-CD8, Mouse mAb, Dako, Cat: M7103, Clone: 144B
anti-Phospho-p44/42 MAPK (Erk1/2) (Thr202/Tyr204) Alexa Fluor® 488 Conjugate Mouse mAb, Cell signaling, Cat: #4374, Clone: (E10)
Anti-Phospho-p38 MAPK (Thr180/Tyr182) PE Conjugate, Rabbit mAb, Cell signaling, Cat: #6908, Clone: (E10)
Anti-PD1, Rabbit mAb, Abcam, Cat: ab137132, Clone: EPR4877(2)
Anti-Ki67, Mouse, mAb, Dako, Cat: M7240, Clone: MIB-1
Anti-Panck, Mouse, mAb, Dako, Cat: M35153, Clone: AE1/AE3

Validation

All antibodies used in the study were validated by the manufacture. Data is available at the manufacture's website as it is indicated below:

anti-EpCAM, Mouse mAb, BioLegend, Cat: 32402, Clone: 9C4: https://www.biolegend.com/en-gb/products/purified-anti-human-cd326-epcam-antibody-3755?GroupID=BLG5134
anti-CD66b, Mouse mAb, BD Biosciences, Cat: 555723, Clone: G10F5: https://www.bdbiosciences.com/en-us/products/reagents/flow-cytometry-reagents/research-reagents/single-color-antibodies-ruo/purified-mouse-anti-human-cd66b.555723
anti-CD11b, Rabbit mAb, BioSB, Cat: BSB6440, Clone: EP45. https://www.biosb.com/biosb-products/cd11b-antibody-rmab-ep45/
anti-CD4, Rabbit mAb, CellMarque, Cat: 104R-26 (AC-0173), Clone: EP204. https://www.cellmarque.com/antibodies/CM/2186/CD4_EP204
anti-CD8, Mouse mAb, Dako, Cat: M7103, Clone: 144B. https://www.agilent.com/en/product/immunohistochemistry/antibodies-controls/primary-antibodies/cd8-(concentrate)-76631
anti-Phospho-p44/42 MAPK (Erk1/2) (Thr202/Tyr204) Alexa Fluor® 488 Conjugate Mouse mAb, Cell signaling, Cat: #4374, Clone: (E10) https://www.cellsignal.com/products/antibody-conjugates/phospho-p44-42-mapk-erk1-2-thr202-tyr204-e10-mouse-mab-alexa-fluor-488-conjugate/4374
Anti-Phospho-p38 MAPK (Thr180/Tyr182) PE Conjugate, Rabbit mAb, Cell signaling, Cat: #6908, Clone: (E10) https://www.cellsignal.com/products/antibody-conjugates/phospho-p38-mapk-thr180-tyr182-3d7-rabbit-mab-pe-conjugate/6908
Anti-PD1 antibody , Abcam, Cat: (ab137132) Clone: [EPR4877(2)] https://www.abcam.com/pd1-antibody-epr48772-ab137132.html
Anti-Ki67, Mouse, mAb, Dako, Cat: M7240, Clone: MIB-1 https://www.agilent.com/en/product/immunohistochemistry/antibodies-controls/primary-antibodies/ki-67-antigen-(dako-omnis)-76239
Anti-Panck, Mouse, mAb, Dako, Cat: M35153, Clone: AE1/AE3, https://www.agilent.com/en/product/immunohistochemistry/antibodies-controls/primary-antibodies/cytokeratin-(concentrate)-76562

Eukaryotic cell lines

Policy information about cell lines

Cell line source(s)	HT-29, HCT-116 and CT26WT were purchased from the American Type Culture Collection (ATCC)<br>HL60 cells expressing GFP-firefly luciferase construct were kindly given by Prof. Cameron Turtle (originally from the ATCC)
Authentication	None of the cell lines used were authenticated.
Mycoplasma contamination	Mycoplasma testing was performed independently by the Research cell bank facility at the Fred Hutch using the MycoProbe

Mycoplasma contamination	Mycoplasma Detection Kit (R&D systems) that can detect the 16S ribosomal RNA of the most common strains of mycoplasma. All cell lines used in this study tested negative for Mycoplasma.
Commonly misidentified lines (See ICLAC register)	No commonly misidentified cell lines were used in the study

Human research participants

Policy information about studies involving human research participants

Population characteristics	All patients included in this analysis were adults diagnosed with either colorectal adenocarcinoma or oral cavity squamous cell carcinoma. A major criteria for inclusion of specimens in this study was that patients were treatment naive at the time of tumor surgical resection. Gender, age or ethnicity of patients were not an exclusion factor as we did not perform analyses based on patient metadata.
Recruitment	All CRC specimens in this study were obtained from bio-repositories and no patients were recruited for this study. The fresh OSCC specimens were obtained from patients with oral cavity squamous cell carcinoma at the University of Washington medical center. These patients had consented to an excess specimen protocol for research, whereby tissue that was obtained under this protocol did not impact patient treatment or outcome. A criteria for patient selection was that they were treatment naive at the time of surgical resection and HPV negative. Age, gender or ethnicity did not impact selection criteria as these factors did not impact our analysis.
Ethics oversight	The use of patient specimens for this work was approved by the Fred Hutchinson Cancer Center IRB under the following protocol numbers RG #: 1006552, 1006974

Note that full information on the approval of the study protocol must also be provided in the manuscript.

Flow Cytometry

Plots

Confirm that:

☒ The axis labels state the marker and fluorochrome used (e.g. CD4-FITC).

☒ The axis scales are clearly visible. Include numbers along axes only for bottom left plot of group (a 'group' is an analysis of identical markers).

☒ All plots are contour plots with outliers or pseudocolor plots.

☒ A numerical value for number of cells or percentage (with statistics) is provided.

Methodology

Sample preparation	For intracellular staining, a total of 10000 cells were incubated LIVE/DEAD fixable Aqua Dead cell staining for 30min on ice. Following incubation, the cells were fixed for 30min and permeabilize using the BD Cytofix/Cytoperm plus kit. The permeabilization solution contained a cocktail of antibodies against phosphoproteins including 1.25µg/ml Phospho-p44/42 MAPK (Erk1/2) (Thr202/Tyr204) (E10) Mouse mAb (Alexa Fluor 488 conjugate) and 1.25µg/ml Phospho-p38 MAPK (Thr180/Tyr182) (3D7) Rabbit mAb (Phycoerythrin conjugate). Following 45min of antibody incubation the cells were washed twice with the permeabilization solution. Cells were resuspended in 200µl FACS wash buffer and 1x104 viable cells (LIVE/DEAD aqua negative cells) were acquired on the BD Fortessa x20 flow cytometer. A tube only containing F. nucleatum was also acquired to gate-out undesired bacteria cell events from the analysis. The geometric mean fluorescence intensity (MFI) was calculated for each phosphoprotein and for each experimental condition relative to an unstained control condition. Flow cytometry data were analyzed with FlowJo software.
Instrument	BD Fortessa x20 flow cytometer
Software	Collection software: BD FACSDiva™ Software<br>Analysis software: FlowJo™ v10.8.1
Cell population abundance	A total of 10000 viable cells events were acquired for each experimental condition
Gating strategy	In all assays, cells were gate for live, single cells and then gated according to the specific staining. A tube only containing F. nucleatum was also acquired to gate-out undesired bacteria cell events from the analysis.

☒ Tick this box to confirm that a figure exemplifying the gating strategy is provided in the Supplementary Information.
